# The impacts of linear infrastructure on terrestrial vertebrate populations: A trait‐based approach

**DOI:** 10.1111/gcb.16450

**Published:** 2022-10-10

**Authors:** Melinda M. J. de Jonge, Juan Gallego‐Zamorano, Mark A. J. Huijbregts, Aafke M. Schipper, Ana Benítez‐López

**Affiliations:** ^1^ Department of Environmental Science, Radboud Institute for Biological and Environmental Sciences (RIBES) Radboud University Nijmegen The Netherlands; ^2^ PBL Netherlands Environmental Assessment Agency The Hague The Netherlands; ^3^ Integrative Ecology Group, Estación Biológica de Doñana Consejo Superior de Investigaciones Científicas (EBD‐CSIC) Sevilla Spain; ^4^ Department of Zoology, Faculty of Sciences University of Granada Granada Spain

**Keywords:** body mass, habitat, herpetofauna, infrastructure‐effect zone, mammals, birds, meta‐analysis, road ecology

## Abstract

While linear infrastructures, such as roads and power lines, are vital to human development, they may also have negative impacts on wildlife populations up to several kilometres into the surrounding environment (infrastructure‐effect zones, IEZs). However, species‐specific IEZs are not available for the vast majority of species, hampering global assessments of infrastructure impacts on wildlife. Here, we synthesized 253 studies worldwide to quantify the magnitude and spatial extent of infrastructure impacts on the abundance of 792 vertebrate species. We also identified the extent to which species traits, infrastructure type and habitat modulate IEZs for vertebrate species. Our results reveal contrasting responses across taxa based on the local context and species traits. Carnivorous mammals were generally more abundant in the proximity of infrastructure. In turn, medium‐ to large‐sized non‐carnivorous mammals (>1 kg) were less abundant near infrastructure across habitats, while their smaller counterparts were more abundant close to infrastructure in open habitats. Bird abundance was reduced near infrastructure with larger IEZs for non‐carnivorous than for carnivorous species. Furthermore, birds experienced larger IEZs in closed (carnivores: ≈130 m, non‐carnivores: >1 km) compared to open habitats (carnivores: ≈70 m, non‐carnivores: ≈470 m). Reptiles were more abundant near infrastructure in closed habitats but not in open habitats where abundances were reduced within an IEZ of ≈90 m*.* Finally, IEZs were relatively small in amphibians (<30 m). These results indicate that infrastructure impact assessments should differentiate IEZs across species and local contexts in order to capture the variety of responses to infrastructure. Our trait‐based synthetic approach can be applied in large‐scale assessments of the impacts of current and future infrastructure developments across multiple species, including those for which infrastructure responses are not known from empirical data.

## Graphical abstract

Linear infrastructures are vital to human development, but they may also have negative impacts on wildlife populations up to several kilometres into the surrounding environment (infrastructure‐effect zones, IEZs). Here, we synthesized 253 studies worldwide to quantify the magnitude and spatial extent of infrastructure impacts on the abundance of 792 vertebrate species. Impacts of infrastructure in mammals were mediated by body size and diet, and depended on the local context (closed vs. open habitat) in mammals, birds and reptiles, yielding IEZs that ranged between 30 and 600 m on average. These differing responses can confound the impact of infrastructure on vertebrate populations when assessments do not account for species traits.

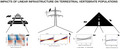

## 1 INTRODUCTION

Linear infrastructures such as roads, railways and power lines are vital to human development and span across a large part of the earth's surface (Dulac, 2013; Meijer et al., 2018). For example, by 2010 there were over 40 million km of paved roads and almost 1 million km of railway tracks globally (Dulac, 2013). Linear infrastructure is expected to significantly increase in the coming decades, especially in developing, biodiversity‐rich nations (Dulac, 2013; Laurance et al., 2014). However, linear infrastructure has documented negative impacts on biodiversity (Benítez‐López et al., 2010; van der Ree et al., 2015). As many future infrastructure projects are planned in some of the world's remaining wilderness areas such as tropical forests (Laurance et al., 2014; Meijer et al., 2018), it is important to synthesize, quantify and understand the impacts of linear infrastructure on biodiversity. This will help not only to evaluate potential negative consequences of future infrastructure developments, but also to design and prioritize mitigation measures.

Infrastructure construction and use affect wildlife through various processes, with habitat destruction and fragmentation, and increased mortality being the most obvious (van der Ree et al., 2015). Additionally, infrastructure use may degrade the surrounding habitat through chemical and noise pollution and the creation of habitat edges (Forman & Alexander, 1998; van der Ree et al., 2015). Furthermore, infrastructure may pose a barrier to species' movement, potentially limiting gene flow between conspecifics and reducing access to important food resources (Forman & Alexander, 1998; Holderegger & Di Giulio, 2010; Skuban et al., 2017; van der Ree et al., 2015). As a result, linear infrastructure may affect wildlife populations up to several kilometres into the surrounding environment. This impact zone is commonly known as the road‐effect zone in road ecology (Forman & Alexander, 1998), and here we expand the term to infrastructure‐effect zone (IEZ) to encompass also other types of infrastructure.

While impacts have been most extensively studied for paved roads, various studies report that wildlife populations are also affected by other infrastructure, including railways and unpaved roads (Barrientos et al., 2019; Benítez‐López et al., 2010; Maynard et al., 2016) as well as power and pipe lines (Biasotto & Kindel, 2018; D'Amico et al., 2018; Richardson et al., 2017). For example, power line collisions and electrocutions have been identified as one of the main avian mortality causes, especially for raptors (Biasotto & Kindel, 2018). However, the impact of different types of infrastructure on the surrounding wildlife may vary, for example, paved roads may have a higher impact on wildlife than unpaved roads because they are often wider and are more intensively used (van der Ree et al., 2015). Infrastructure impacts may further depend on the characteristics of the surrounding habitat. Closed habitats, like forests, are usually more affected by edge effects (Khamcha et al., 2018), while noise and air pollution travel further in open habitats (Benítez‐López et al., 2010; Forman & Alexander, 1998; van der Ree et al., 2015).

Infrastructure impacts also differ between species (Benítez‐López et al., 2010; Rytwinski & Fahrig, 2012; van der Ree et al., 2015). Species with low reproductive rates and long generation times may be disproportionately impacted by infrastructure collisions and mortality as their populations recover more slowly than populations of species with high reproductive rates (Forman et al., 2003; Rytwinski & Fahrig, 2011). Additionally, species with large home ranges are more likely to encounter infrastructure thus increasing the chance of mortality (Forman et al., 2003; Rytwinski & Fahrig, 2011, but see also Pfeifer et al., 2017). As home range size and reproductive rate are related to body size (Hendriks, 2007; Tucker et al., 2014), large species are expected to be disproportionately affected by infrastructure (Rytwinski & Fahrig, 2011, 2012). In contrast, small species may be more abundant in infrastructure verges due to changes in the vegetation or because their larger predators are less abundant (Ascensão et al., 2012; Forman & Alexander, 1998; Ouédraogo et al., 2020; Planillo et al., 2018). Lastly, carnivores may be more affected than herbivores as they generally have larger home ranges and lower reproductive rates (Hendriks, 2007; Tucker et al., 2014). However, some carnivores may be attracted to infrastructure for resources, such as carrion or small mammals that live in the verges (Forman & Alexander, 1998; Morelli et al., 2014; Planillo et al., 2018).

Despite these context dependencies, the impacts of linear infrastructure on biodiversity have so far been assessed based on aggregated biodiversity indicators (Benítez‐López et al., 2010; Torres et al., 2016), or for a limited number of species only (e.g. Andrasi et al., 2021; Carter et al., 2020). These limitations typically result from a lack of data on species‐specific responses to infrastructure rather than a lack of recognition that infrastructure impacts may differ between habitats, infrastructure types and species (Tulloch et al., 2019). While various studies have investigated the interplay between species traits and infrastructure impacts, none have systematically quantified the relationship between species traits and IEZs. Recently, Tulloch et al. (2019) proposed using expert opinions to delineate species‐specific IEZs for various types of infrastructure. However, the plethora of local empirical studies spanning many species from various taxa offers the possibility of employing a trait‐based meta‐analytical approach to quantify the impacts of linear infrastructure across vertebrate species.

Here we quantified the magnitude and spatial extent of linear infrastructure impacts on the abundance of terrestrial vertebrates (mammals, birds, amphibians, reptiles). We performed an extensive meta‐analysis of 253 studies, from 110 primary sources, spanning 160, 443, 97 and 92 species of mammals, birds, reptiles and amphibians respectively. We used meta‐regression models to identify the extent to which species traits (i.e. body size and diet), infrastructure type (i.e. paved roads, unpaved roads, pipelines, power lines) and habitat (i.e. open or closed) modulate infrastructure effects on vertebrate abundance. We hypothesized that large vertebrates are most negatively impacted by infrastructure, with paved roads having a larger impact than unpaved roads, followed by pipe and power lines (Table 1). Additionally, we expected narrower IEZs but with larger abundance declines in closed compared to open habitats. Finally, we expected carnivorous species to be more abundant in the proximity of roads because of their use as feeding grounds (roadkills) or as corridors for dispersal.

**TABLE 1:** Moderators included in our meta‐regression models with expected relationships, background, taxonomic groups for which each moderator is included and source of data source.

Predictor variable	Hypothesis	Terms	Taxonomic group (data source)
Distance (m, log_10_‐transformed)	‐ The negative impacts of infrastructure on the surroundings, such as noise and chemical pollution, decrease with increasing distance from infrastructure (Forman & Alexander, 1998; van der Ree et al., 2015) ‐ Some animals make use of infrastructure corridors which may lead to increased abundances at very close distances (Andersen et al., 2017; Ouédraogo et al., 2020) Expectation: Abundances decrease with increasing distance in the direct vicinity of infrastructure but increase steadily thereafter	Distance + Distance^2^	All (from source)
Diet (% use of vertebrates, fish and scavenging)	‐ Carnivorous mammals and birds reportedly feed on roadkilled carrion (Morelli et al., 2014). Scavenging birds may be able to detect carcasses in infrastructure clearings more easily than those in the habitat interior (Lambertucci et al., 2009). Birds of prey have been spotted perching close to infrastructure before hunting on rodents or other species which are more abundant in the proximity of infrastructure (Morelli et al., 2014). Species with diets consisting of a larger percentage of vertebrates or scavenging may be more likely to venture close to the infrastructure than those with a small percentage of vertebrate use Expectation: Attraction to infrastructure with increasing percentage of diet consisting of vertebrates	Distance × Diet + Distance^2^ × Diet	Birds: (EltonTraits) Mammals: (EltonTraits) Reptiles & Amphibians: Not included
Mean body mass (g, log_10_‐transformed)	‐ Large species have low reproductive rates and may be more negatively impacted by infrastructure as their populations recover slowly from population declines (Rytwinski & Fahrig, 2012) ‐ Larger species have larger home ranges leading them to interact with infrastructure more often which makes them more susceptible to traffic collisions or electrocution from power lines (D'Amico et al., 2018; Rytwinski & Fahrig, 2012) ‐ Infrastructure verges may provide a refuge for small vertebrates (Morelli et al., 2014; Ouédraogo et al., 2020) ‐ Large birds have lower song frequencies which have a higher overlap with the frequency of traffic noise (Francis, 2015) Expectation: Large species are more affected and experience larger effect zones	Distance × BM + Distance^2^ × BM	Mammals: (EltonTraits) Birds: (EltonTraits) Reptiles: (Feldman et al., 2016) Amphibians: (Santini et al., 2018, estimated from snout‐vent‐length)
Infrastructure type 3(4)‐levels ‐ Paved roads ‐ Unpaved roads ‐ Non‐traffic (includes power lines for mammals, reptiles and amphibians) ‐ Power lines (birds only)	‐ Roads have an additional negative impact from traffic disturbance (noise, light, chemical emissions) and traffic mortality (Richardson et al., 2017; van der Ree et al., 2015) ‐ Traffic volume and speed is higher on paved than unpaved roads leading to more disturbance as well as higher traffic mortality (Forman & Alexander, 1998) ‐ Paved road surfaces might be avoided (Ascensão et al., 2016; Brehme et al., 2013; Chen & Koprowski, 2019) ‐ Power lines might add to mortality rates for birds (Biasotto & Kindel, 2018; D'Amico et al., 2018) Expectation: Paved roads have a larger impact than unpaved roads which in turn have a larger impact than non‐traffic infrastructure such as seismic lines, pipeline and power lines	InfraType	All (from source)
Habitat 2‐levels ‐ Open ‐ Closed	‐ Noise and chemical pollution travel further in open habitats potentially increasing the width of the infrastructure‐effect zone (van der Ree et al., 2015) ‐ Edge effect may be more pronounced in closed habitats leading to larger effects close to the infrastructure (van der Ree et al., 2015) Expectation: Negative impact near infrastructure is larger in closed habitats, but effect‐zone is wider in open habitats	Distance × Habitat + Distance^2^ × Habitat	All (from source)

## 2 METHODS

### 2.1 Data collection

#### 2.1.1 Literature search

We collated data from studies included in a previous meta‐analysis on infrastructure impacts on biodiversity (Benítez‐López et al., 2010) and complemented it by searching for additional data in peer‐reviewed literature in the ISI Web of Science and Google Scholar in April 2020 using the following search terms: (vertebrate* OR *bird* OR *fauna OR reptil* OR lizard* OR snake* OR turtle* OR tortoise* OR crocodil* OR amphibia* OR frog* OR toad* OR salamander* OR mammal*) AND (infrastruct* OR road$ OR motorway* OR highway* OR “train track” OR railway* OR “transmission line” OR power$line* OR “seismic line” OR pipeline*) AND (disturbance* OR effect* OR impact* OR distance* OR proximity OR avoidance OR influence) AND (density OR abundan* OR encounter$ OR population$ OR count$ OR persistence). We also used ProQuest Dissertations and Theses repository (https://www.proquest.com/products‐services/dissertations/) and Open Access Theses and Dissertations repository (https://oatd.org/) to search for additional grey literature. We also used a ‘snowball’ method, in which we reviewed the references of all included papers to identify additional relevant studies based on their title and whether they were cited in a context that suggested they collected data on infrastructure impacts on vertebrates. While we used an English‐based literature search, studies in Spanish (Delgado et al., 2004; Vargas‐Salinas et al., 2011), German (Ballasus & Sossinka, 1997) and Portuguese (Bager & da Rosa, 2012) retrieved from the original dataset by (Benítez‐López et al., 2010) or via cross‐referencing were also included. Full details of the literature search and modifications of the search string to the specifications of each database can be found in the Supporting Information (S1 Search Strategy).

#### 2.1.2 Inclusion criteria

After an initial screening based on title and abstract, we selected publications that met the following inclusion criteria:

- The authors reported on the effect of linear infrastructure on nearby populations of birds, mammals, reptiles or amphibians.
- The authors reported abundances or densities at species or genus level.
- The authors reported abundances or densities from at least one site close to the infrastructure and one undisturbed and more distant site. Alternatively, the authors reported abundances at several distances from the infrastructure (with a minimum of two distances), where the one furthest away is considered a control, undisturbed site.

The initial title and abstract screening was done by MMJdJ, AB‐L and MAJH. When in doubt about the relevance of a particular study, the authors discussed among each other to reach consensus about its transfer to the next screening phase. Full‐text screening and data extraction was split between JG‐Z and MMJdJ. We evaluated inter‐observer agreement between the two screeners by calculating Cohen's kappa (*κ*) based on a random selection of 50 sources (*κ* = .92).

The search string and database from Benítez‐López et al. (2010) yielded 5794 unique publications from which we selected 809 publications based on the title and abstract. We added an additional five sources through cross‐referencing (see Figure S1 for a PRISMA flow diagram showing the screening process). Based on full‐text screening we selected 110 publications published between 1979 and 2020 for data extraction. A list of all data sources is provided in the Supporting Information.

#### 2.1.3 Data extraction

We structured the data into data source (i.e. publication), study and species, where a single data source may contain one or more studies, depending on whether data are reported for one or more infrastructure types or distinct locations. A study contains one or more species for which abundances are reported at least in one site close by infrastructure (disturbed), and one site further away or in a designated control area (control) in relation to a specific infrastructure. Paired sites (disturbed and control) were always reported within the same study, had similar biophysical (habitat) characteristics, and reported species abundance using the same sampling method.

From each study, we extracted the mean abundance of each species at each distance from the infrastructure, the standard deviation of the mean abundance and the sample size. We extracted the data from text and tables when possible, or from graphs using WebPlotDigizer (https://automeris.io/WebPlotDigitizer/). When the study reported medians instead of means or range or interquartile range instead of the standard deviation, we calculated the mean and standard deviation following Wan et al. (2014). Abundances were reported as various metrics including: number of individuals, population density (individuals/ha), group density (groups/ha), trapping rates (individuals per trapping effort), dropping or scat density (scats/ha), nest densities (nests/ha) or territory density (territories/ha). When abundances were reported in distance intervals we took the middle distance point of the interval as the input distance.

We also extracted the following study characteristics: type of infrastructure (e.g. dirt road, secondary paved road, highway, power line, seismic line, pipeline, logging tracks), habitat (e.g. grassland, cropland, shrubland, tropical forest, temperate forest), location (continent, country), geographical coordinates (longitude, latitude) and year(s) in which the empirical data collection was done. We classified the type of infrastructure into (i) paved roads (including highways), (ii) unpaved roads (dirt roads, gravel roads) and (iii) non‐traffic infrastructure (trails, seismic lines, pipelines, power lines). For birds, we included power lines as a separate category as we expect an additional impact from power line collisions compared to other non‐traffic infrastructure (Biasotto & Kindel, 2018). We classified habitat type into open (grasslands, croplands, shrublands) and closed (forests). If geographical coordinates were not provided by the authors, we retrieved them by geo‐referencing maps or descriptions of the study area in the paper using Google Earth.

**FIGURE 1:**
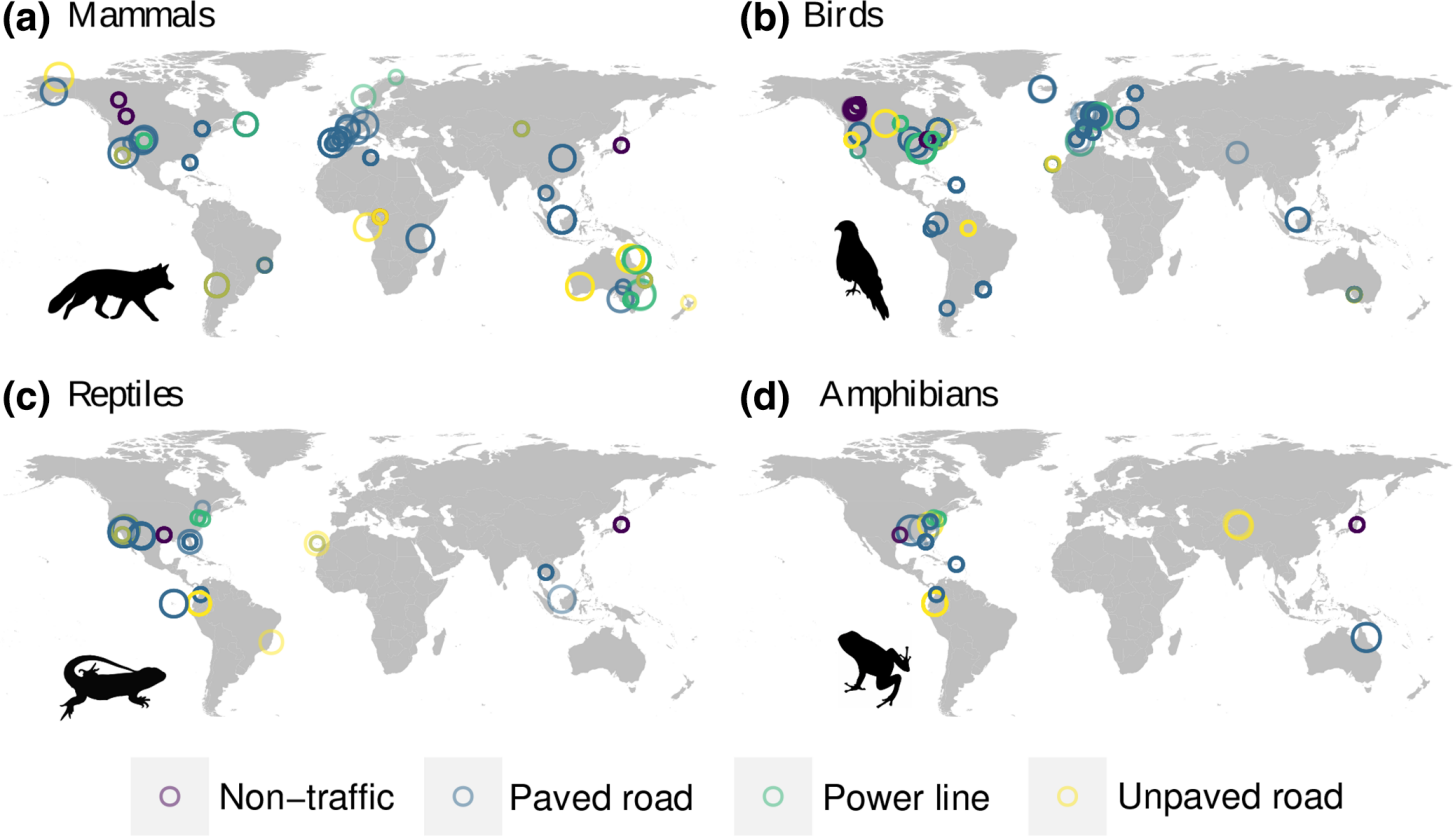
Spatial distribution of studies included in our meta‐analysis for mammals (a), birds (b), reptiles (c) and amphibians (d). Size of points is proportional to the number of species included in each study. Power lines are shown separately but were combined with non‐traffic infrastructure for mammals, reptiles and amphibians in all analyses. Silhouettes are public domain obtained from ‘phylopic’ (www.phylopic.org).

Our final database contained 3912 pairwise abundance comparisons between disturbed and non‐disturbed areas distributed across 26 countries and six continents (Figure 1). Of these, 863 comparisons were for mammals (160 species in 17 orders and 38 families), 2471 for birds (443 species in 22 orders and 88 families), 362 for reptiles (97 species in 2 orders and 22 families) and 216 for amphibians (92 species in 2 orders and 16 families). Distance to infrastructure ranged from 0 to 4500 m for mammals, from 0 to 3485 m for birds, from 0 to 1600 m for reptiles and from 0 to 120 m for amphibians. The majority of the comparisons came from paved roads (53% of all comparisons) and closed habitats (69% of all comparisons; we found no data from open habitats for amphibians; see Tables S1–S4 for an overview of the number of effect sizes per habitat and infrastructure type).

#### 2.1.4 Species traits

We gathered data on mean body mass (g) and diet (% of diet consisting of vertebrates, fish or scavenging) for mammals and birds from the EltonTraits database (Wilman et al., 2014). We calculated the mean body mass for amphibians from mean snout–vent length (SVL) using the allometric relationships developed by Santini et al. (2018). We extracted mean SVL from Santini et al. (2018) and AmphibiaWeb (AmphibiaWeb, 2016; https://amphibiaweb.org). We obtained mean body mass of reptiles from allometric relationships with SVL or total length (Feldman et al., 2016). When abundances were given on the genus level (e.g. *Cephalophus* sp.) or were aggregated for multiple species within a genus (e.g. *Felis sylvestris* and *F. catus*), we calculated the mean body mass and diet (% of diet consisting of vertebrates, fish or scavenging) across all species in the genus or aggregated group (9% of mammals, <1% of birds).

Body masses in our database ranged from 3.6 to 3.9 × 10^6^ g for mammals, 4.3 to 1.1 × 10^4^ g for birds, 0.7 to 3.5 × 10^4^ g for reptiles and 1.4 × 10^−3^ to 3.1 × 10^2^ g for amphibians (Figure S2). Most mammal and bird species in our database were non‐carnivorous, that is, with 0% of the diet consisting of other vertebrates or scavenging (63% of mammals and 73% of birds) and only 8% of mammals and 6% of birds had a diet consisting of ≥80% vertebrates, fish or scavenging (Figure S3). While per cent diet is a continuous variable in our models, we present most of our results for non‐carnivorous and carnivorous species as defined above.

### 2.2 Effect size

For each study *i*, species *s* and distance from infrastructure *d*, we calculated the effect size as the natural logarithm of the response ratio (LRR_*isd*_), that is, the logarithm of the ratio of the mean abundance at the affected site (*Ā*_*isd*_) and the mean abundance at the control site (*Ā*_*isc*_). Because many of the included studies had a small sample size, we applied a small sample bias correction to the effect sizes following the Delta method (LRR^∆^; Lajeunesse, 2015):

(1) $${\mathrm{LRR}}_{\mathrm{isd}}^{∆}=\log\left(\frac{{\bar{A}}_{\mathrm{isd}}}{{\bar{A}}_{\mathrm{isc}}}\right)+\frac{1}{2}\left(\frac{{\mathrm{SD}}_{\mathrm{isd}}^{2}}{n_{\mathrm{isd}}{\bar{A}}_{\mathrm{isd}}^{2}}-\frac{{\mathrm{SD}}_{\mathrm{isc}}^{2}}{n_{\mathrm{isc}}{\bar{A}}_{\mathrm{isc}}^{2}}\right),$$

where ${\mathrm{SD}}_{\mathrm{isd}}^{2}$ is the sampling variance of the mean abundance at the affected site and ${\mathrm{SD}}_{\mathrm{isc}}^{2}$ is the sampling variance of the mean abundance in the control site. Effect sizes are therefore negative (LRR^Δ^ <0) or positive (LRR^Δ^ >0) if abundance estimates are lower or higher, respectively, near infrastructure. In some cases, a species was not detected in areas close to the infrastructure or the control area, precluding calculation of the effect size. To circumvent this, we used the truncated sample means (*Ã*) following Pustejovsky (2015):

(2) $$\tilde{A}=\left\{\begin{array}{cc} \bar{A}\; & \text{if}\;\bar{A}>0\\ 1/\left(2\mathrm{nD}\right) & \text{if}\;\bar{A}=0 \end{array}\right.$$

where *n* is the sample size of the corresponding mean and *D* corrects for the scale on which the abundance is reported (e.g. *D* is equal to 1 when outcomes are reported as total number of individuals averaged over *n* samples while *D* is equal to the number of trap nights when outcomes are reported as number of individuals per trap night averaged over *n* samples). Treatment or control mean was equal to zero for 20%, 19%, 20% and 26% of the comparisons for mammals, birds, reptiles and amphibians respectively.

Observed effect sizes (LRR^∆^) were weighed by the inverse of their corresponding sampling variances, which were calculated as:

(3) $$\mathrm{VAR}\left({\mathrm{LRR}}_{\mathrm{isd}}^{∆}\right)=\frac{{\mathrm{SD}}_{\mathrm{isd}}^{2}}{n_{\mathrm{isd}}{\tilde{A}}_{\mathrm{isd}}^{2}}+\frac{{\mathrm{SD}}_{\mathrm{isc}}^{2}}{n_{\mathrm{isc}}{\tilde{A}}_{\mathrm{isc}}^{2}}+\frac{1}{2}\left(\frac{{\mathrm{SD}}_{\mathrm{isd}}^{4}}{n_{\mathrm{isd}}^{2}{\tilde{A}}_{\mathrm{isd}}^{4}}+\frac{{\mathrm{SD}}_{\mathrm{isc}}^{4}}{n_{\mathrm{isc}}^{2}{\tilde{A}}_{\mathrm{isc}}^{4}}\right).$$

When no variance estimate was reported, or if the reported variance was equal to zero, we estimated it by assuming that the data follow a Poisson distribution so that *Ã* = SD^2^ (22%, 63%, 26%, and 54% of response ratios for mammals, birds, reptiles and amphibians respectively).

### 2.3 Analysis

#### 2.3.1 Overall impacts of infrastructure

We ran four multilevel random‐effects meta‐analyses to estimate the overall impact of infrastructure (regardless of its proximity or any context‐dependent factors or species traits) on mammal, bird, reptile and amphibian abundances respectively. We included observation ID as a random effect ($\sigma_{1}^{2}$) to account for residual heterogeneity. We also included species ($\sigma_{3}^{2}$) nested in order or family ($\sigma_{2}^{2}$) and study ($\sigma_{5}^{2}$) nested in source ($\sigma_{4}^{2}$) as random effects to account for non‐independence of response ratios and assess variance between sources, studies, orders, families and species. We included order for mammals and birds because body mass is conserved within orders for these groups (Böhning‐Gaese & Oberrath, 1999; Smith et al., 2004). For reptiles and amphibians, there were too few orders, two for each group, to include as random effects term and body mass can vary substantially between families within the same order (Mesquita et al., 2016; Phung et al., 2020). To control for non‐independence due to multiple treatments per study sharing the same control, we used the full variance–covariance matrix in our analysis following Gleser and Olkin (2009) and Lajeunesse (2011). We assessed residual heterogeneity of the meta‐analyses using the weighted least squares extension of Cochran's *Q*‐test (*Q*_E_).

#### 2.3.2 Influence of distance, habitat type, infrastructure type and species traits

We first examined the relationship between the LRR^∆^ and distance to infrastructure and derived overall IEZs (distance to infrastructure where the modelled LRR^∆^ = 0) for each of the four species groups using single mixed‐effects meta‐regressions. We log_10_‐transformed distance to infrastructure and included it as both a linear and a quadratic term to account for possible non‐linear responses. Next, we ran multiple mixed‐effects meta‐regressions for each taxonomic group to examine variations in the relationship between LRR^∆^ and distance while controlling for the effects of habitat type, infrastructure type, body size (g, log_10_‐transformed) and diet (% of diet consisting of vertebrates, fish or scavenging, only for mammals and birds). For body size, diet and habitat we also included interaction terms with distance as we expected that the relationship between infrastructure impacts and distance are modulated by these moderators (see details in Table 1). We included infrastructure type only as main effect (see details in Table 1). Prior to the analysis, we tested for collinearity between body mass and diet for mammals (Spearman *ρ* = .07, Figure S3a) and birds (Spearman *ρ* = .56, Figure S3b). For each taxon we selected the most parsimonious model based on the Akaike information criterion (AICc) calculated from the full log‐likelihood (Verbyla, 2019). For the selected models, we evaluated model fit by calculating the marginal and conditional explained heterogeneity ($R_{m}^{2}$ and $R_{c}^{2}$; Nakagawa & Schielzeth, 2013) and quantified the amount of heterogeneity explained by the moderators using the omnibus test (*Q*_M_). We also tested main effects of individual moderators and interactions with *Q*_M_, where interaction terms were dropped to test the main effects. Lastly, we checked profile likelihood plots to ensure the identifiability of the variance components ($\sigma_{1}^{2}$: observation‐level variability, $\sigma_{2}^{2}$: order or family‐level variability, $\sigma_{3}^{2}$: species‐level variability, $\sigma_{4}^{2}$: source‐level variability, $\sigma_{5}^{2}$: study‐level variability; Figures S4–S7). Models are deemed non‐identifiable when there is more than one likely parametrization of the variance components, which results in a multimodal or flat profile (Raue et al., 2009).

Results are reported as LRR^∆^, percentage abundance change (percentage change = (exp(LRR^∆^) − 1) × 100) or width of IEZ. All models were fitted with REML (restricted maximum likelihood) using the package metafor v3.0‐2 in R4.0.1 (Viechtbauer, 2010). We used ggplot2 v3.3.3 (Wickham et al., 2016) and pals v1.7 (Wright, 2021) for data visualization and foreach v1.5.1 (Wallig, 2020a) and doParallel v1.0.16 (Wallig, 2020b) to run models in parallel for model selection.

### 2.4 Robustness of results

#### 2.4.1 Publication bias

We assessed publication bias using Funnel plots and Egger tests for each species group (Egger et al., 1997). We performed Egger tests by modelling meta‐analytic residuals as a function of precision (1/SE) and extracting the modelled intercept.

#### 2.4.2 Sensitivity to small sample means

The LRR^∆^ is sensitive to cases where the mean abundance of either the affected or control site is near zero (Lajeunesse, 2015). Therefore, we tested the robustness of our results to small sample means by selecting LRR^∆^ for which the small‐sample corrected standardized mean of both the control and infrastructure site passed Geary's rule (Lajeunesse, 2015):

(4) $$\frac{\tilde{A}}{\mathrm{SD}}\;\left(\frac{4n^{3/2}}{1+4n}\right)\geq 3.$$

This selection reduced the number of effect sizes to 279 (33% of effect sizes) for mammals, 943 (38%) for birds, 140 (39%) for reptiles and 63 (29%) for amphibians. We compared the results of the random‐effects meta‐analysis using the selected data set to the results obtained from the complete database.

#### 2.4.3 Imputation of sampling variance

To test the robustness of our results to the imputation of missing sampling variances we also imputed missing SD using the ‘Bracken1992’ and the ‘HotDeckNN’ approach and compared the random‐effects meta‐analysis results with our default approach for imputing missing SD (‘Poisson’). With the ‘Bracken1992’ approach, missing SDs are estimated from the SD to mean ratio from all studies with complete information (Bracken, 1992; Lajeunesse, 2013). The ‘HotDeckNN’ approach uses Rubin and Schenker's (1991) resampling approach to fill missing SD with SD of studies with complete information that have a similar mean (Lajeunesse, 2013). For the ‘HotDeckNN’ approach we imputed missing SD 100 times leading to 100 meta‐analytical estimates. We also compared the results from the three imputation methods with the results from the subset of the data for which SDs were available. We used the package metagear v0.7 (Lajeunesse, 2016) to impute standard deviations based on the ‘Bracken1992’ and ‘HotDeckNN’ approach.

#### 2.4.4 Study quality

To test the sensitivity of our results to the quality of the data sources we assigned each effect size quality score based on the following criteria:

- The authors reported abundances on the species level or these could be derived from the raw data (1 point). The authors reported abundance on the genus level (0 points).
- The authors reported abundances in undisturbed sites or the authors reported abundances at a distance from the infrastructure that was equal to or larger than the home range of the species as reported by the authors (1 point). The largest distance between a sampling site and the infrastructure was smaller than the reported home range of the species or the home range of the species was not specified (0 points).

We repeated our random‐effects meta‐analysis three times: once while including all effect sizes, once excluding effect sizes based on abundances aggregated over multiple species (*a* = 0, mammals: 1%, birds: 4%, reptiles: 0%, amphibians: 0%) and once excluding effect sizes for which the control site was not explicitly defined as undisturbed or at larger distances from infrastructure than the species' home range (*b* = 0, mammals: 64%, birds: 89%, reptiles: 56%, amphibians: 66%).

## 3 RESULTS

### 3.1 Overall effects of infrastructure

We found evidence of decline in species abundance across all infrastructure sites when compared to the corresponding control sites for amphibians, but not for mammals, birds and reptiles (LRR^∆^ [95% CI] of mammals: 0.07 [−0.15 to 0.30]; birds: −0.13 [−0.33 to 0.06]; reptiles: −0.03 [−0.22 to 0.17]; amphibians: −0.22 [−0.41 to −0.02]). Cochran's *Q* (*Q*_E_) indicated significant residual heterogeneity for all species groups (Table S5). When we removed LRR^∆^ with small sample means our results were similar to those from the full database but had larger confidence intervals (Table S6). We found no evidence of publication bias for any of the four species groups (Figure S8). Mean response ratios were similar for all imputation approaches (Table S7), and when studies were excluded based on the quality criteria (Figure S9).

### 3.2 Influence of distance to infrastructure

**FIGURE 2:**
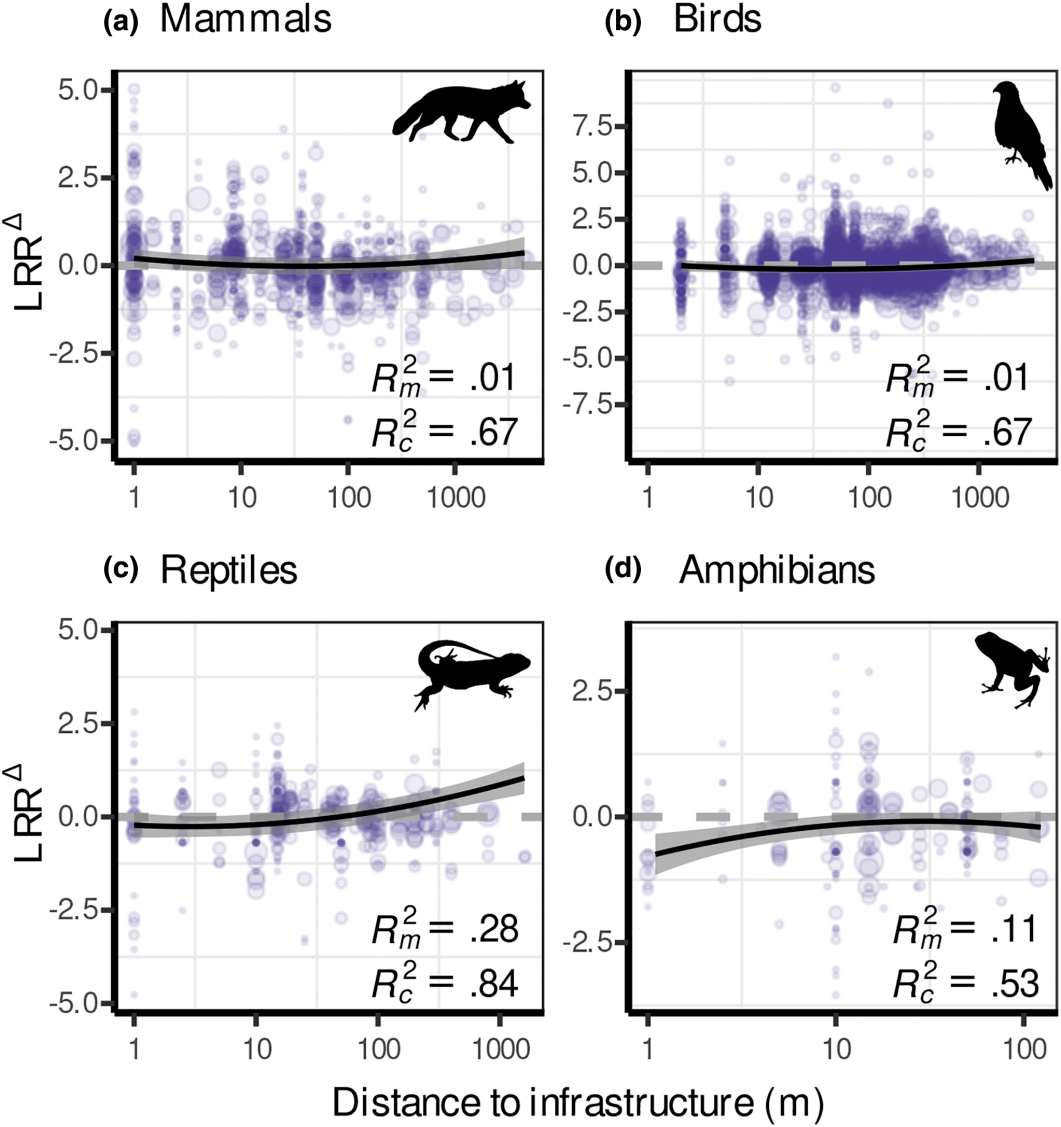
Change in species abundance (LRR^∆^, natural logarithm of response ratio between abundance at infrastructure site and abundance at control site) as a function of distance to infrastructure for mammals (a), birds (b), reptiles (c) and amphibians (d). Models were fitted using multilevel meta‐regression models with distance to infrastructure (log_10_‐transformed, including quadratic term for birds and reptiles) as moderator and observation ID, source ID, study ID (nested in source ID), order ID (or family ID for reptiles and amphibians) and species ID (nested in order or family) as random effects. Size of data points is proportional to the natural logarithm of the inverse of the sampling variance. Grey bands represent the 95% CI of the modelled relationship between LRR^∆^ and distance to infrastructure. LRR^∆^ < 0 indicates abundance decline, LRR^∆^ > 0 indicates abundance increase and LRR^∆^ = 0 indicates no change (dashed grey line). Silhouettes are public domain obtained from ‘phylopic’ (www.phylopic.org).

Across species, habitats and infrastructure types, we found that the observed LRR^∆^ were non‐linearly related to distance to infrastructure (log_10_‐transformed) in all four species groups (Figure 2; Table S5). Mammal abundances were increased by 23% at 1 m from infrastructure, declined to −2% within the first 40 m, and then increased again leading to IEZs of 105 m (Figure 2a; Table S5). Similarly, bird abundances were increased by 11% at 1 m from infrastructure and declined to −18% over the first 35 m with an overall IEZ of 655 m (Figure 2b; Table S5). Reptile abundances followed a similar pattern but were reduced by 20% near infrastructure with an IEZ of 48 m (Figure 2c; Table S5). Abundances of amphibians were reduced by about −52% at a distance of 1 m to infrastructure, but increased rapidly with distance from infrastructure, yielding an IEZ of 27 m (Figure 2d; Table S5).

### 3.3 Influence of species traits, infrastructure type and habitat characteristics

**FIGURE 3:**
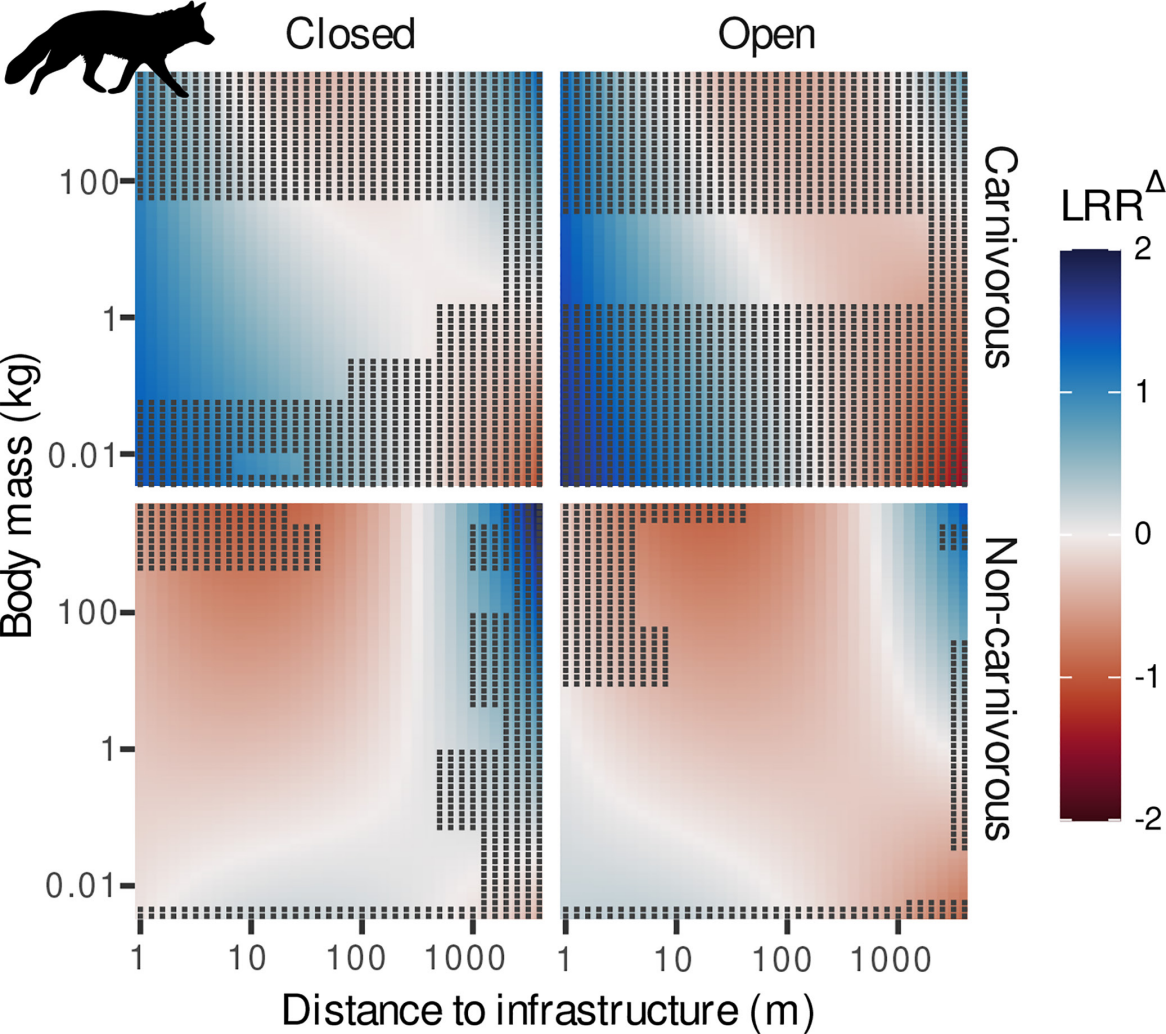
Change in species abundance (LRR^∆^) as a function of distance to infrastructure and body mass for carnivorous (upper panels, 80% of diet from vertebrates or scavenging) and non‐carnivorous mammals (lower panels, 0% of diet from vertebrates or scavenging) in closed (left panels) and open habitats (right panels) based on the best model (see Table 2 for parameter estimates and *R* ^2^ values). Plots show the predicted LRR^∆^ due to infrastructure from the final model as colour gradient ranging from blue (abundance increase, LRR^∆^ > 0) to red (abundance decrease, LRR^∆^ < 0) where white represents no change in abundance (LRR^∆^ = 0). Dots indicate extrapolation areas. Silhouettes are public domain obtained from ‘phylopic’ (www.phylopic.org).

IEZs varied across species based on their body mass and diet, and according to the environmental context (infrastructure type and habitat type). The moderators of infrastructure impacts differed among the four species groups (Table 2; Tables S8–S11). Infrastructure impacts on mammal abundances were best explained by body mass, diet and habitat, and their interactions with distance to infrastructure, which collectively accounted for 9% of the heterogeneity in the observations (Table 2; Table S8). Further heterogeneity was attributed to differences between species (49%) and data sources (15%; Table S12). As expected, the percentage of vertebrates, fish and scavenging in the diet was positively related to abundance responses in mammals. Carnivorous mammals (80% of diet consisting of vertebrates, fish and scavenging) were more abundant near infrastructure and became less abundant at larger distances (Figure 3). The positive effect of infrastructure on carnivorous mammals was more apparent for small‐sized carnivores than for large‐sized carnivores and persisted over larger distances (small carnivores: 107 m, large carnivores: 21 m), with little variation between close and open habitats. In turn, abundance responses of non‐carnivorous (0% vertebrates, fish and scavenging) mammals varied between habitat types and body size, with small‐sized species (<1 kg) having higher abundance in the proximity of infrastructure in open habitats, and larger species having reduced abundances in both open and closed habitat types. IEZs varied between 2 and 603 m for small‐sized and large‐sized non‐carnivores, respectively, with variations among habitat types (Table 3).

**TABLE 2:** Parameter estimates plus 95% confidence intervals and *p*‐values of AICc selected meta‐regression models for mammals, birds, reptiles and amphibians. Distance: Distance to infrastructure (m, log_10_‐transformed), BM: Mean body mass (g, log_10_‐transformed), habitat (open/closed, categorical), InfraType (paved road/power line/unpaved road/non‐traffic, categorical). Cochran's *Q* test for residual heterogeneity (*Q*_E_), omnibus test of moderators (*Q*_M_), marginal explained variance ($R_{m}^{2}$) and conditional explained ($R_{c}^{2}$) are given for the model. Omnibus tests are also performed for each of the moderators where main effects were tested after dropping interactions.

	Moderator	Estimate (95% CI)	*p*_estimate_	*Q*_M_ (df)	*p*_QM_
Mammals					
*Q*_M,9_ = 74 (*p* < .0001)	Intercept	−0.00 (−0.45, 0.45)	.994		
*Q*_E,853_ = 6029 (*p* < .0001)	Distance	0.45 (0.02, 0.88)	.040	5.92 (2)	.052
$R_{m}^{2}$ = .09, $R_{c}^{2}$ = .75	Distance^2^	−0.16 (−0.32, −0.02)	.030		
	Diet	0.02 (0.01, 0.03)	<.001	9.71 (1)	.002
	BM	−0.08 (−0.26, 0.10)	.377	1.41 (1)	.236
	Habitat(open)	0.29 (−0.05, 0.63)	.095	0.29 (1)	.591
	Distance × Diet	−0.01 (−0.01, −0.00)	<.001	23.82 (1)	<.001
	Distance × BM	−0.20 (−0.37, −0.03)	.020	23.67 (2)	<.001
	Distance^2^ × BM	0.09 (0.04, 0.14)	<.001		
	Distance × Habitat(open)	−0.23 (−0.37, −0.08)	.002	9.34 (1)	.002
Birds					
*Q*_M,8_ = 62 (*p* < .001)	Intercept	0.05 (−0.32, 0.42)	.795		
*Q*_E,2462_ = 26,188 (*p* < .001)	Distance	−0.04 (−0.13, 0.04)	.302	6.65 (1)	.010
$R_{m}^{2}$ = .04, $R_{c}^{2}$ = .66	Diet	−0.01 (−0.02, −0.01)	.001	1.43 (1)	.232
	Habitat(open)	−0.47 (−0.87, −0.06)	.024	3.59 (1)	.058
	InfrastructureType(paved road)	−0.45 (−0.80, −0.10)	.011	6.63 (3)	.085
	InfrastructureType(power line)	−0.29 (−0.70, 0.13)	.172		
	InfrastructureType(unpaved road)	−0.32 (−0.75, 0.12)	.151		
	Distance × Diet	0.01 (0.01, 0.01)	<.001	23.44 (1)	<.001
	Distance × Habitat(open)	0.34 (0.20, 0.54)	<.001	18.84 (1)	<.001
Reptiles					
*Q*_M,4_ = 64 (*p* < .001)	Intercept	0.18 (−0.27, 0.63)	.432		
*Q*_E,357_ = 1335 (*p* < .001)	Distance	−0.44 (−0.87, −0.00)	.048	57.30 (2)	<.001
$R_{m}^{2}$ = .31, $R_{c}^{2}$ = .84	Distance^2^	0.15 (0.03, 0.28)	.025		
	Habitat(open)	−0.67 (−1.25, −0.08)	.017	0.19 (1)	.661
	Distance × Habitat(open)	0.38 (0.09, 0.67)	.009	6.81 (1)	.009
Amphibians					
*Q*_M,2_ = 9 (*p* = .013)	Intercept	−0.74 (−1.15, −0.33)	.000		
*Q*_E,213_ = 1407 (*p* < .001)	Distance	0.91 (0.21, 1.62)	.011	8.74 (2)	.013
$R_{m}^{2}$ = .11, $R_{c}^{2}$ = .53	Distance^2^	−0.32 (−0.64, 0.00)	.051		

**TABLE 3:** Infrastructure‐effect zones (IEZ) plus 95% confidence intervals estimated by final models (Table 2) for small (10th percentile of body masses), medium and large (90th percentile of body masses) reptiles, carnivorous (80% of diet consisting of vertebrates, fish or scavenging) mammals and birds and non‐carnivorous (0% of diet consisting of vertebrates, fish or scavenging) birds and mammals. IEZ estimates for birds correspond to paved roads. ^+^Indicates a positive effect of infrastructure on the abundance within the IEZ. No IEZ was calculated for non‐carnivorous birds in open habitats as the modelled response ratios did not increase as a function of distance to infrastructure. See also Figure S11 for a continuous representation of IEZs as a function of diet and body mass for mammals

Class	Diet	Body mass	Open IEZ (m) (95% CI)	Closed IEZ (m) (95% CI)
Mammals	Carnivore	Small (100 g)	68 (24, 1840)^+^	107 (31, −)^+^
Mammals	Carnivore	Medium (3000 g)	36 (10, −)^+^	47 (9, −)^+^
Mammals	Carnivore	Large (30,000 g)	21 (6, −)^+^	22 (4, −)^+^
Mammals	Non‐carnivore	Small (10 g)	76 (0, 5203)^+^	2 (0, −)
Mammals	Non‐carnivore	Medium (2000 g)	1 (0, 11)^+^	224 (39, 1303)
Mammals	Non‐carnivore	Large (400,000 g)	603 (167, 1870)	295 (79, 892)
Birds	Carnivore	—	67 (34, 132)	130 (46, 491)
Birds	Non‐carnivore	—	469 (75, 6590)	—
Reptiles	—	—	92 (10, 368)	3 (0, −)^+^
Amphibians	—	—	27 (7, −)	27 (7, −)

**FIGURE 4:**
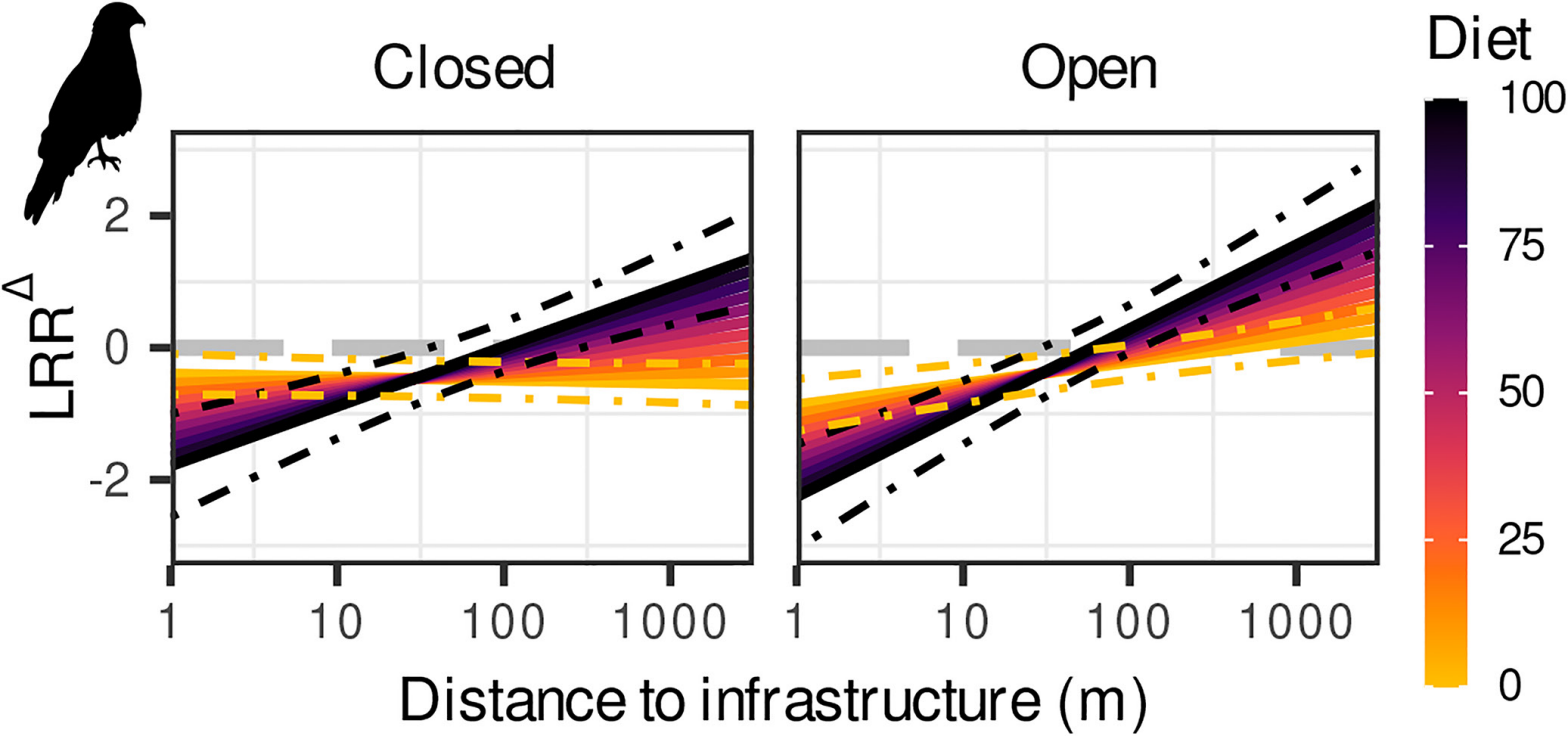
Change in species abundance (LRR^∆^) as a function of distance to infrastructure and diet (% of diet consisting of vertebrates or scavenging, indicated by colour) for birds in closed (left panel) and open (right panel) habitats based on the best model (see Table 2 for parameter estimates and *R* ^2^ values). Dashed lines represent the 95% confidence interval for 0% and 100% of diet consisting of vertebrates or scavenging. LRR^∆^ < 0 indicates abundance decline, LRR^∆^ > 0 indicates abundance increase and LRR^∆^ = 0 indicates no change (dashed grey line). Silhouettes are public domain obtained from ‘phylopic’ (www.phylopic.org).

Infrastructure impacts on birds were modulated by diet, habitat type and infrastructure type and followed a linear relationship with the log_10_ transformed distance to infrastructure (Table 2; Table S9). Fixed effects explained only 4% of the heterogeneity between observed effect sizes with further variation attributed to differences between studies (30%), species (22%) and orders (9%; Table S12). Bird abundances were lower near paved roads than near unpaved roads and power lines, and were highest near other non‐traffic infrastructure (seismic lines, pipelines, trails; Table 2; Figure S10). Furthermore, carnivorous birds experienced larger abundance reductions but smaller IEZs than non‐carnivorous birds. Likewise, abundance reductions near infrastructure were higher in open habitats but extended over shorter distances compared to closed habitats. In closed habitats, abundances declined to −78% and −33% for carnivorous and non‐carnivorous birds respectively. The corresponding IEZ for carnivorous birds was 130 m while no IEZ could be calculated for non‐carnivores. In turn, abundances of carnivorous birds in open habitats were reduced by −86% with an IEZ of 67 m while non‐carnivorous birds were reduced by −58% with an IEZ of 469 m (Figure 4; Table 3).

**FIGURE 5:**
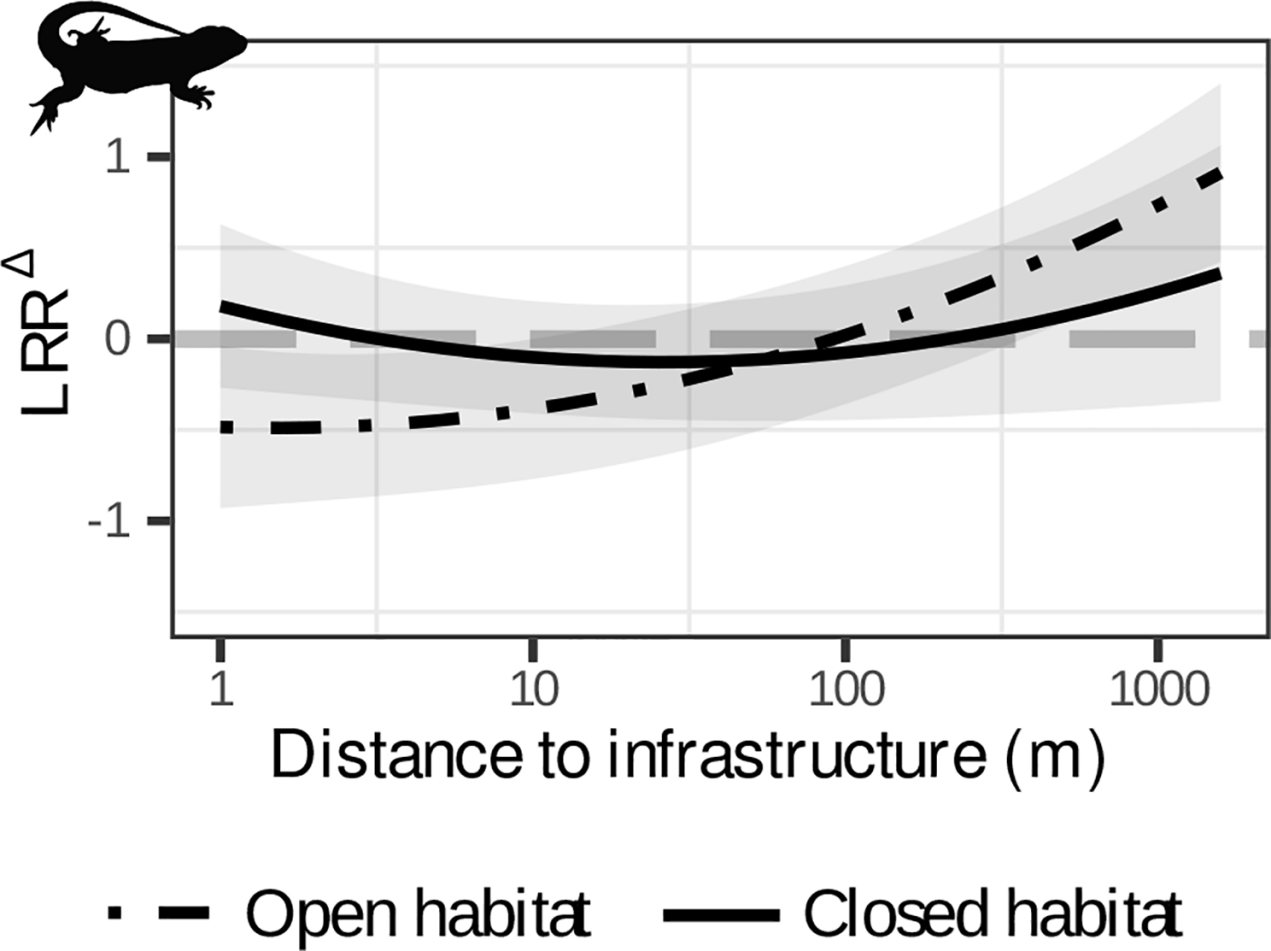
Change in species abundance (LRR^∆^) as a function of distance to infrastructure for reptiles in closed (solid line) and open (dot‐dashed line) habitats based on the best model (see Table 2 for parameter estimates and *R* ^2^ values). Grey bands represent the 95% CI of the modelled relationship between LRR^∆^ and distance to infrastructure. LRR^∆^ < 0 indicates abundance decline, LRR^∆^ > 0 indicates abundance increase and LRR^∆^ = 0 indicates no change (dashed grey line). Silhouettes are public domain obtained from ‘phylopic’ (www.phylopic.org).

For reptiles, the relationship between LRR^∆^ and distance to infrastructure was only modulated by habitat type, which explained about 31% of the heterogeneity (Table 2; Table S10). Additional heterogeneity in response ratios was attributed to differences between species (27%) and data sources (25%; Table S12). In closed habitats, abundance ratios of reptiles increased with +20% near infrastructure compared to control areas and decrease to 0% in the first 5 m (Figure 5). In contrast, reptile abundances in open habitats were reduced by −39% near infrastructure, with an IEZ of 92 m (Table 3). Lastly, amphibian responses were not related to body mass or infrastructure type. Instead, only distance to infrastructure and its quadratic term were retained in the final model (Figure 2d; Table 2; Table S11). However, the variance components of the model suggest a large variability between individual species (42%; Table S12).

## 4 DISCUSSION

The development of linear infrastructure is pervasive across the globe and is one of the main drivers of global change. Yet, the impacts of linear infrastructure on species abundance have not been comprehensively quantified. Here we contribute to our understanding of the impacts of linear infrastructure on terrestrial vertebrates by synthesizing the findings from local studies across the world. We performed a meta‐analysis of reported changes in species‐specific abundance in the proximity of infrastructure for mammals, birds, reptiles and amphibians. However, as infrastructure impacts vary between species and environmental context, across‐species averages may not give an adequate representation of IEZs and changes in population abundance. To address this, we used a trait‐based approach to reveal general functional responses to infrastructure applicable to a wide range of species. We also accounted for differences in environmental characteristics, such as habitat type and infrastructure type. We found that infrastructure impacts were modulated by diet (birds and mammals), body size (mammals), habitat type (birds, mammals and reptiles) and infrastructure type (birds).

Our results yield a relatively small average IEZ for mammals (≈100 m) which is substantially lower than previous estimates (5 km, Benítez‐López et al., 2010). These diverging results likely stem from the use of different biodiversity metrics. Benítez‐López et al. (2010) employed an aggregated biodiversity indicator (mean species abundance) which was truncated so that positive responses (increased abundances) were not included. Our meta‐regression indicates that infrastructure effects on mammal populations are more nuanced and depend on both biological traits and the environmental context. We report contrasting responses of carnivorous and non‐carnivorous mammals, with the former being more abundant near infrastructure, whereas the latter consistently display avoidance responses. Indeed, carnivores of varying body sizes are reportedly observed in the vicinity of roads because they are attracted to roadkill carcasses or because they use infrastructure verges as movement corridors (Andersen et al., 2017; Planillo et al., 2018). Avoidance responses by non‐carnivorous mammals depend on their body size and the type of habitat. Medium‐ to large‐sized non‐carnivorous mammal species were more affected over larger distances than smaller species. Because medium‐ and large‐sized species usually have larger home ranges than small‐sized species, they may encounter infrastructure more frequently (Rytwinski & Fahrig, 2012; Tucker et al., 2014). Here we show that these responses may be exacerbated in open habitats (IEZ ≈600 m) where visibility is higher than in closed habitats (IEZ ≈300 m). If infrastructure is perceived as a threat similar to predation risk (Frid & Dill, 2002), we would expect that animals in open habitats, where anti‐predator vigilance is more effective, forage at larger distances from infrastructure than in closed habitats, where higher alertness and quick fleeing responses might be more beneficial (Duffett et al., 2020). Small‐sized species, in turn, have smaller home ranges and may even use infrastructure verges as a habitat or refuge from predators (Ascensão et al., 2012; Ouédraogo et al., 2020), particularly in open habitats (Table 3).

Birds were generally more negatively affected by infrastructure than mammals, leading to an average IEZ of about 650 m, which is similar to recently reported road‐effect zones in Great Britain (500–700 m, Cooke et al., 2020). Paved roads had the largest impact, followed by power lines and other non‐traffic infrastructure. Previous evidence has linked high traffic volumes and traffic speed on paved roads to high levels of chemical and noise pollution, as well as traffic mortality (van der Ree et al., 2015), which have detrimental effects on birds (noise: Grade & Sieving, 2016, roadkills: Grilo et al., 2020). Variation between bird species' responses was related to diet, with carnivores experiencing smaller IEZs (≈100 m) than non‐carnivorous birds (≈470 m). While raptors were less abundant near infrastructures, their small IEZ might indicate that areas near infrastructure may act as complementary hunting grounds due to the potential increase of small mammals (Lambertucci et al., 2009). Furthermore, our results indicate that the impact of linear infrastructures is greater in closed compared to open habitats. Species in closed habitats tend to have lower frequency vocalizations than those in open habitats where low‐frequency sounds degrade faster (Boncoraglio & Saino, 2007), and may be thus more impacted by low‐frequency traffic noise due to greater spectral overlap (Francis, 2015; Francis et al., 2011). Finally, in contrast to our expectations, we did not find a relationship between mean body size and infrastructure impacts (Tables 1 and 2). This might be explained by the overrepresentation of passerines in our database, 83% and 51% of all observations in closed and open habitats respectively. Passerines, which are generally small birds, heavily rely on vocal communications with high song complexity, making them especially sensitive to traffic noise (Catchpole & Slater, 2003; Francis et al., 2011).

We provide the first estimates of the IEZ across many species of reptiles. Reptile responses were highly variable with positive responses in closed habitats but negative responses in open habitats. The derived IEZs in open habitats (≈90 m) were smaller but of the same order as those previously reported for single species (e.g. Tanner & Perry, 2007: 200 m, *Microlophus albemarlensis*; Peaden et al., 2015: 203–306 m, *Gopherus agassizii*). In closed habitats, reptiles may use infrastructure sites for thermoregulation because of increased ground temperatures and solar radiation due to clearings in the forest canopy (Meiri et al., 2013; Sullivan, 1981; Tuff et al., 2016). While we expected that large reptiles would be more affected by infrastructure, we found no evidence of infrastructure impacts being modulated by body mass. This might be explained by opposing relationships between biological traits that affect reptile sensitivity to infrastructure and body size. Previous evidence indicate that thermal biology may play an important role in modulating ectotherm responses to infrastructure, which increases surface temperatures and lowers humidity in the surrounding habitat (Tuff et al., 2016; van der Ree et al., 2015). Reptile sensitivity to infrastructure may therefore decrease with optimal body temperature, which is in turn positively related to body size (Meiri et al., 2013; Nowakowski et al., 2018). Similar considerations may explain our findings for amphibians. While small amphibians generally have faster life histories, they are also more sensitive to dehydration and have lower critical body temperatures than larger‐bodied species (Liu et al., 2021; Nowakowski et al., 2018; Pfeifer et al., 2017; Tracy et al., 2010). Our results for amphibians should, however, be interpreted with caution as the maximum distance to infrastructure (120 m), as well as the average distance of the control sites (200 m), is smaller than previously reported amphibian IEZs, which are however based on distributional data and habitat preferences instead of abundance estimates (e.g. Eigenbrod et al., 2009: 600–1000 m; Hamer et al., 2021: 1000 m).

A considerable proportion of heterogeneity in our analysis was related to differences between species, suggesting other species traits may explain variability in infrastructure impacts. Examples of traits that may be included if data become available are call frequency and the potential to shift vocal frequencies (birds and amphibians; Francis, 2015; Liu et al., 2021), larval habitat (amphibians; Liu et al., 2021), migratory status (all species groups; Beebee, 2013; Cooke et al., 2020; Grilo et al., 2020; Southwood & Avens, 2010), foraging behaviour (e.g. active or ambush predation for reptiles and ground, understory, aerial or aquatic for birds; Francis, 2015; Meiri et al., 2013) and habitat guild (e.g. aquatic, fossorial, terrestrial or arboreal; reptiles and amphibians; Meiri et al., 2013; Tracy et al., 2010). Another interesting research direction would be to investigate species traits that affect how well species adapt to anthropogenic environments such as habitat specificity and relative brain size (Fristoe et al., 2017; Keinath et al., 2017; Liu et al., 2021; Santini et al., 2019). Furthermore, for mammals, birds and amphibians, a considerable amount of heterogeneity was captured by the source‐ and study‐level random effects, indicating a role for study design specifications or location‐specific factors. For example, mortality rates as well as chemical and noise pollution scale with traffic intensity and speed (van der Ree et al., 2015). Similarly, climatic conditions such as wind speed and direction, affect how hydrological, chemical and noise impact travel into the surrounding environment (van der Ree et al., 2015). Yet, such information is not consistently reported and could therefore not be included in our analysis.

Besides changes in abundance, alternative metrics such as mortality rates, demographic rates and gene flow between populations may provide additional insight into the processes that underlie infrastructure impacts (Ascensão et al., 2016; González‐Suárez et al., 2018; Holderegger & Di Giulio, 2010; Teixeira et al., 2020). For example, attraction to roads may lead to increased mortality rates in carnivorous mammals and reptiles with active foraging strategies (González‐Suárez et al., 2018; Sosa & Schalk, 2016). As a result, our reported positive responses to infrastructure for carnivores may translate into reduced long‐term population persistence if collision rates in an area exceed the population growth rate (Ceia‐Hasse et al., 2017; Grilo et al., 2021; Planillo et al., 2018). Additionally, easily measurable responses, such as roadkills, may be collected in citizen‐science projects and can provide valuable information for species and geographical locations that are under‐represented in population‐level studies (Périquet et al., 2018; Valerio et al., 2021).

While we have performed the most comprehensive synthesis study on infrastructure impacts on species abundances of terrestrial vertebrates, our study has some limitations. As it is commonly the case in ecology and biodiversity research (Hughes et al., 2021; Martin et al., 2012), the majority of the studies in our dataset originate from North America and Western Europe, and we found no studies originating from Southern and Western Asia and Eastern Europe. As a result, species endemic to those regions were not included in our analysis, while many future infrastructure projects are planned in those areas (Laurance et al., 2015; Meijer et al., 2018). This geographical bias is, to some extent, a reflection of our English‐language‐based literature search (Amano et al., 2021). Future studies addressing global infrastructure impacts on biodiversity should aim at including search strings in other languages to include more non‐English literature to cover currently under‐represented regions and species (Amano et al., 2021; Barrientos et al., 2021; Konno et al., 2020). Furthermore, most of the studies we included use a control‐impact or space‐for‐time design, which is arguably less robust for capturing changes in biodiversity than before–after control‐impact (BACI) studies (Christie et al., 2019). Yet, we prioritized maximizing sample size and taxonomic representativeness over having a few BACI studies focused on a limited number of species and locations. Finally, while we only compared paired disturbed and control sites with similar habitat types and vegetation characteristics, we cannot fully dismiss potential confounding effects of site differences resulting from, for example, past land use or natural spatial species turnover. The latter is, however, less likely at the spatial scale of our analyses, which involve pairwise comparisons within a few hundred metres. Both of these limitations are common in other space‐for‐time meta‐analyses and are impossible to assess without having long‐term studies with the same study design as the one reported in the primary studies.

To our knowledge, this is the first study to perform a meta‐analysis of changes in abundance in proximity to infrastructure and IEZs using species‐specific abundances for mammals, birds, reptiles and amphibians. Our results suggest that infrastructure impacts are highly variable between species and habitat contexts, which should be taken into account in multi‐species infrastructure assessments. So far, most large‐scale assessments of the current infrastructure network have used a generic IEZ for all species (Ibisch et al., 2016; Schipper et al., 2020; Torres et al., 2016). Instead, our models can be applied across species and habitats by accommodating variable IEZs based on species traits and the distribution of habitat and infrastructure types within their geographical range. Such an approach may also reflect how the composition of a community could change in terms of individual species as well as functional diversity. Moreover, our results can be used to assess the impacts on biodiversity of planned infrastructure projects. Massive expansions of the global infrastructure network are expected within the next decades to give access to traffic and energy‐related infrastructure in poor and currently disconnected areas following UN Sustainable Development Goals 7 and 9 (SDG7, SDG9; Fuso Nerini et al., 2018; Thacker et al., 2019). However, because many of these developments are planned in key areas for biodiversity they are in conflict with SDG15, which aims for halting land degradation and biodiversity loss (Baste et al., 2021; Narain et al., 2020; Thacker et al., 2019). The most prominent example, China's Belt Road Initiative, potentially intersects up to 1500 key biodiversity areas across Asia, Europe and Africa (Li & Shvarts, 2017; Narain et al., 2020). These areas can be important habitats for already endangered species including rhinoceros, orangutans, elephants and tigers (Alamgir et al., 2019; Carter et al., 2020). Similar concerns exist for infrastructure developments in other regions, such as South‐America (Ascensão et al., 2022; Laurance et al., 2015). Quantitative assessments of the impacts of future infrastructure on biodiversity are the first step in re‐aligning SDG9 with SDG15 by informing spatial planning and mitigation and compensation policies (Milner‐Gulland et al., 2021; zu Ermgassen et al., 2019). However, these assessments should also consider indirect effects of new infrastructure such as increased hunting pressure in newly accessible areas, habitat fragmentation, land encroachment and exotic‐species invasions (Benítez‐López et al., 2017, 2019; Ceia‐Hasse et al., 2017; Laurance & Arrea, 2017; Liu et al., 2019; Torres et al., 2016). Only with a holistic approach that encompasses the myriad impacts of infrastructure on wildlife we may be able to effectively tackle the loss of biodiversity linked to current and future infrastructure networks.

## AUTHOR CONTRIBUTIONS

Ana Benítez‐López developed the first ideas for this manuscript. Ana Benítez‐López, Aafke M. Schipper, Mark A. J. Huijbregts and Melinda M. J. de Jonge designed the research. Ana Benítez‐López, Juan Gallego‐Zamorano, Mark A. J. Huijbregts and Melinda M. J. de Jonge were involved in the first screening of the literature. Juan Gallego‐Zamorano and Melinda M. J. de Jonge conducted the full‐text screening of the literature and extracted the data. Melinda M. J. de Jonge performed the data analysis. All authors contributed to the interpretation and discussion the results. Melinda M. J. de Jonge wrote the first draft of the manuscript and all authors contributed meaningfully to revisions of it.

## CONFLICT OF INTEREST

The authors declare that they have no competing interests.

## Supporting information


Appendix S1.

## Data Availability

The data that support the findings of this study are openly available in DANS‐EASY at https://doi.org/10.17026/dans‐xcw‐zyvh. The scripts of the analyses underlying the results of this study, as well as the final fitted models for further application in environmental impact assessments, are available at https://github.com/MelindadeJonge/Infrastructure_MetaAnalysis.
