# Supplementary material for: The impacts of linear infrastructure on terrestrial vertebrate populations: A trait‐based approach
Source: Glob Chang Biol. 2022 Oct 10;28(24):7217–33. doi: 10.1111/gcb.16450 (PMC9827953; doi:10.1111/gcb.16450)
Supplement: Supplementary file 1 — Appendix S1. [file GCB-28-7217-s001.docx]

### Supplementary information to:

# The impacts of linear infrastructure on terrestrial vertebrate populations: a trait-based approach

Melinda M.J. de Jonge^1*^, Juan Gallego-Zamorano^1^, Mark A.J. Huijbregts^1^, Aafke M. Schipper^1,2^, Ana Benítez-López^1,3,4^

^1^ Department of Environmental Science, Radboud Institute for Biological and Environmental Sciences (RIBES), Radboud University, Nijmegen, The Netherlands

^2^ PBL Netherlands Environmental Assessment Agency, The Hague, The Netherlands

^3^Integrative Ecology Group, Estación Biológica de Doñana, Consejo Superior de Investigaciones Científicas (EBD-CSIC), Sevilla, Spain

^4^Department of Zoology, Faculty of Sciences, University of Granada, Granada, Spain

* Corresponding author: [m.dejonge@fnwi.ru.nl](mailto:m.dejonge@fnwi.ru.nl), phone: +31 (0)24 365 32 81

Melinda M.J. de Jonge – ORCID: 0000-0003-4760-8155

Juan Gallego-Zamorano – ORCID: 0000-0003-2317-5148

Mark A.J. Huijbregts – ORCID: 0000-0002-7037-680X

Aafke M. Schipper – ORCID: 0000-0002-5667-0893

Ana Benítez-López – ORCID: 0000-0002-6432-1837

## S1 Search Strategy

### Web of Science

We searched ISI Web of Science in April 2020 using the following search string in the field “Topic (TS)”:

TS=((vertebrate* OR *bird* OR *fauna OR reptil* OR lizard* OR snake* OR turtle* OR tortoise* OR crocodil* OR amphibia* OR frog* OR toad* OR salamander* OR mammal*) AND (infrastruct* OR road$ OR motorway* OR highway* OR "train track" OR railway* OR “transmission line” OR power$line* OR “seismic line” OR pipeline*) AND (disturbance* OR effect* OR impact* OR distance* OR proximity OR avoidance OR influence) AND (density OR abundan* OR encounter$ OR population$ OR count$ OR persistence))

### Google Scholar

We searched Google Scholar in April 2020 using a modification of the original search string, as Google Scholar does not allow wild cards or Boolean terms. We split the original search string into eight substrings and removed wildcards:

- (reptile|lizard|snake|turtle|tortoise|crocodile)(infrastructure|road|motorway|highway|"train track"|railway)(disturbance|effect|impact|distance|proximity|avoidance|influence)(density|abundance|encounter|population|count|persistence)
- (reptile|lizard|snake|turtle|tortoise|crocodile)(“transmission line”|”power line”| “seismic line”|pipeline)(disturbance|effect|impact|distance|proximity|avoidance|influence)(density|abundance|encounter|population|count|persistence)
- (amphibian|frog|toad|salamander)(infrastructure|road|motorway|highway|"train track"|railway)(disturbance|effect|impact|distance|proximity|avoidance|influence)(density|abundance|encounter|population|count|persistence)
- (amphibian|frog|toad|salamander)(“transmission line”|”power line”| “seismic line”|pipeline)(disturbance|effect|impact|distance|proximity|avoidance|influence)(density|abundance|encounter|population|count|persistence)
- (bird|mammal)(infrastructure|road|motorway|highway|"train track"|railway)(disturbance|effect|impact|distance|proximity|avoidance|influence)(density|abundance|encounter|population|count|persistence)
- (bird|mammal)(“transmission line”|”power line”| “seismic line”|pipeline)(disturbance|effect|impact|distance|proximity|avoidance|influence)(density|abundance|encounter|population|count|persistence)
- (avifauna|herpetofauna|vertebrate)(“transmission line”|”power line”| “seismic line”|pipeline)(disturbance|effect|impact|distance|proximity|avoidance|influence)(density|abundance|encounter|population|count|persistence)
- (avifauna|herpetofauna|vertebrate)(infrastructure|road|motorway|highway|"train track"|railway)(disturbance|effect|impact|distance|proximity|avoidance|influence)(density|abundance|encounter|population|count|persistence)

We collected the first 100 hits for each search string for title and abstract screening.

### ProQuest Dissertations and Theses repository

We searched all fields except full text with the following search string:

(vertebrate* OR bird* OR avifauna OR herpetofauna OR reptil* OR lizard* OR snake* OR turtle* OR tortoise* OR crocodil* OR amphibia* OR frog* OR toad* OR salamander* OR mammal*) AND (infrastruct* OR road OR roads OR motorway* OR highway* OR "train track" OR railway* OR “transmission line” OR power*line* OR “seismic line” OR pipeline*) AND (disturbance* OR effect* OR impact* OR distance* OR proximity OR avoidance OR influence) AND (density OR abundan* OR encounter* OR population OR population* OR count OR counts OR persistence)

### Open Access Theses and Dissertations

We searched “Any field” with the same search string as used in ProQuest:

(vertebrate* OR bird* OR avifauna OR herpetofauna OR reptil* OR lizard* OR snake* OR turtle* OR tortoise* OR crocodil* OR amphibia* OR frog* OR toad* OR salamander* OR mammal*) AND (infrastruct* OR road OR roads OR motorway* OR highway* OR "train track" OR railway* OR “transmission line” OR power*line* OR “seismic line” OR pipeline*) AND (disturbance* OR effect* OR impact* OR distance* OR proximity OR avoidance OR influence) AND (density OR abundan* OR encounter* OR population OR population* OR count OR counts OR persistence)

### Supplementary Figures


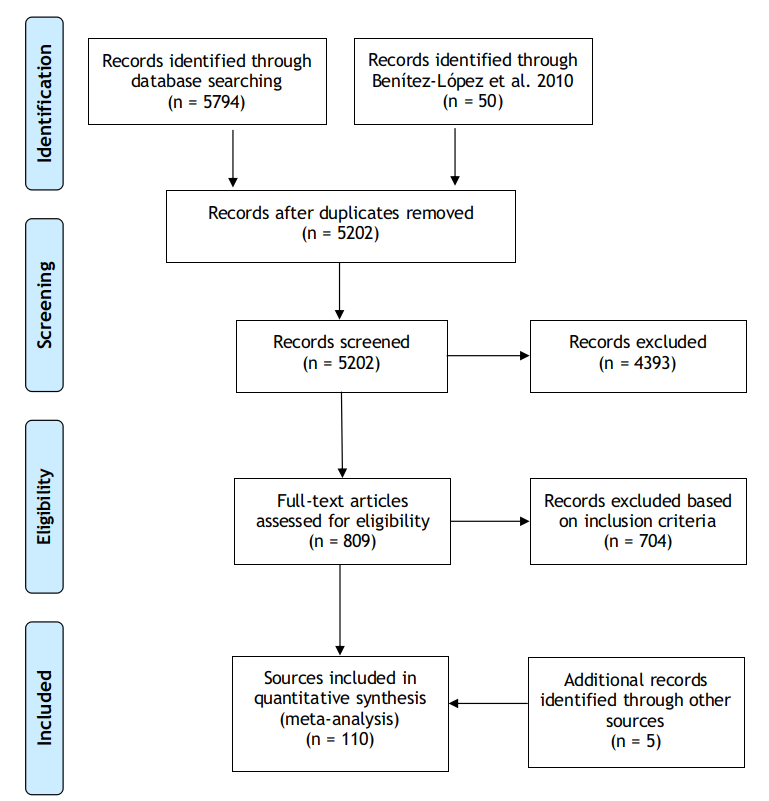


Figure S1: PRISMA flow-chart showing the number of primary sources identified during our literature search and the number of sources retained included during the first and second screening and the final number of primary sources included in the analysis. From the final 110 sources, 30 were obtained from Benítez-López et al. 2010.


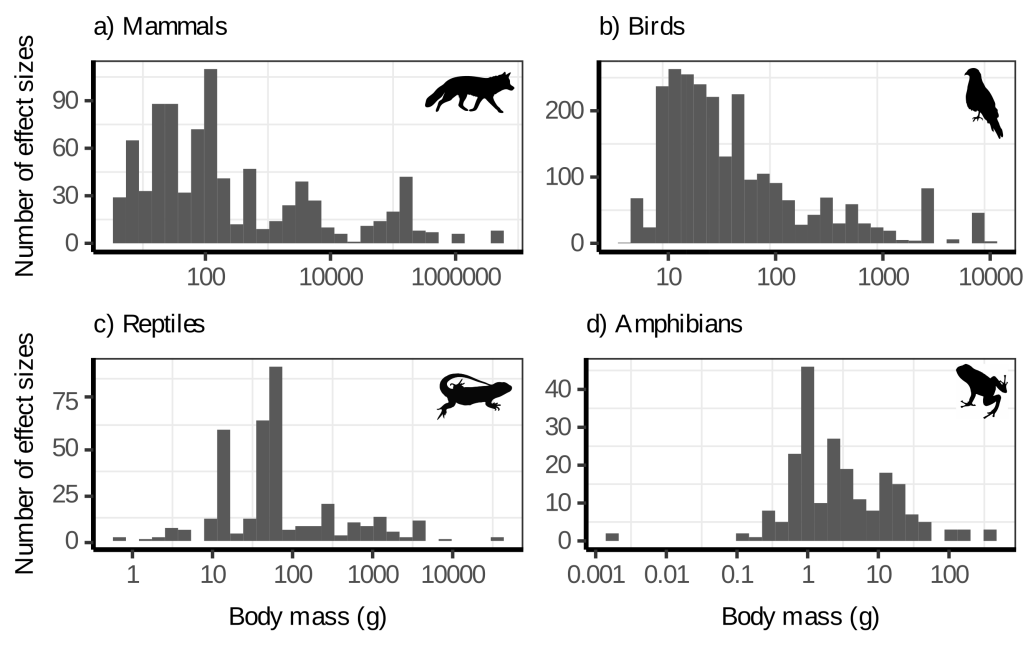


Figure S2: Distribution of mean species body masses (grams) of the species included in our analysis for a) mammals, b) birds, c) reptiles and d) amphibians.


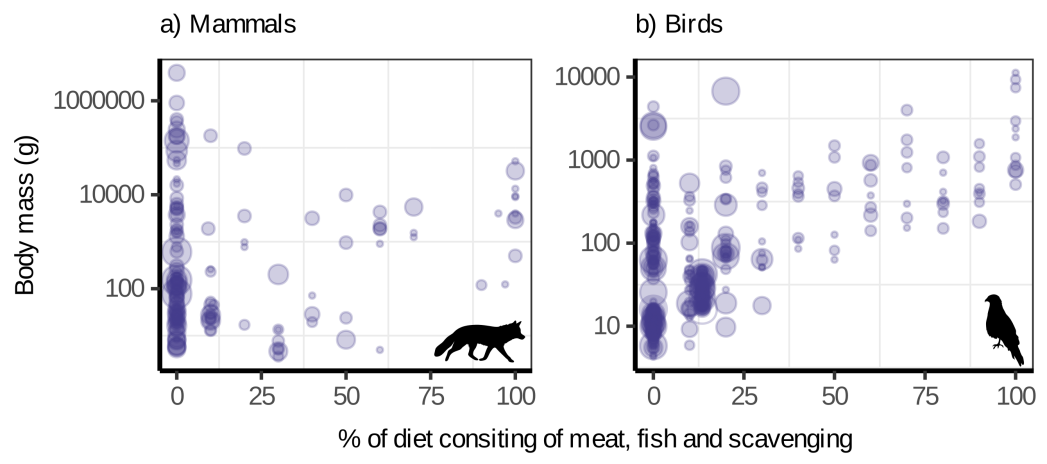


Figure S3: Distribution of mean species body masses (grams) and diet of the species included in our analysis for A) mammals and B) birds. Size of points indicate the number of effect sizes for each species. Spearman rank correlation between body mass and diet is for mammals lower (Spearman *rho* = 0.07, n=160) than for birds (Spearman *rho* = 0.56, n=443).


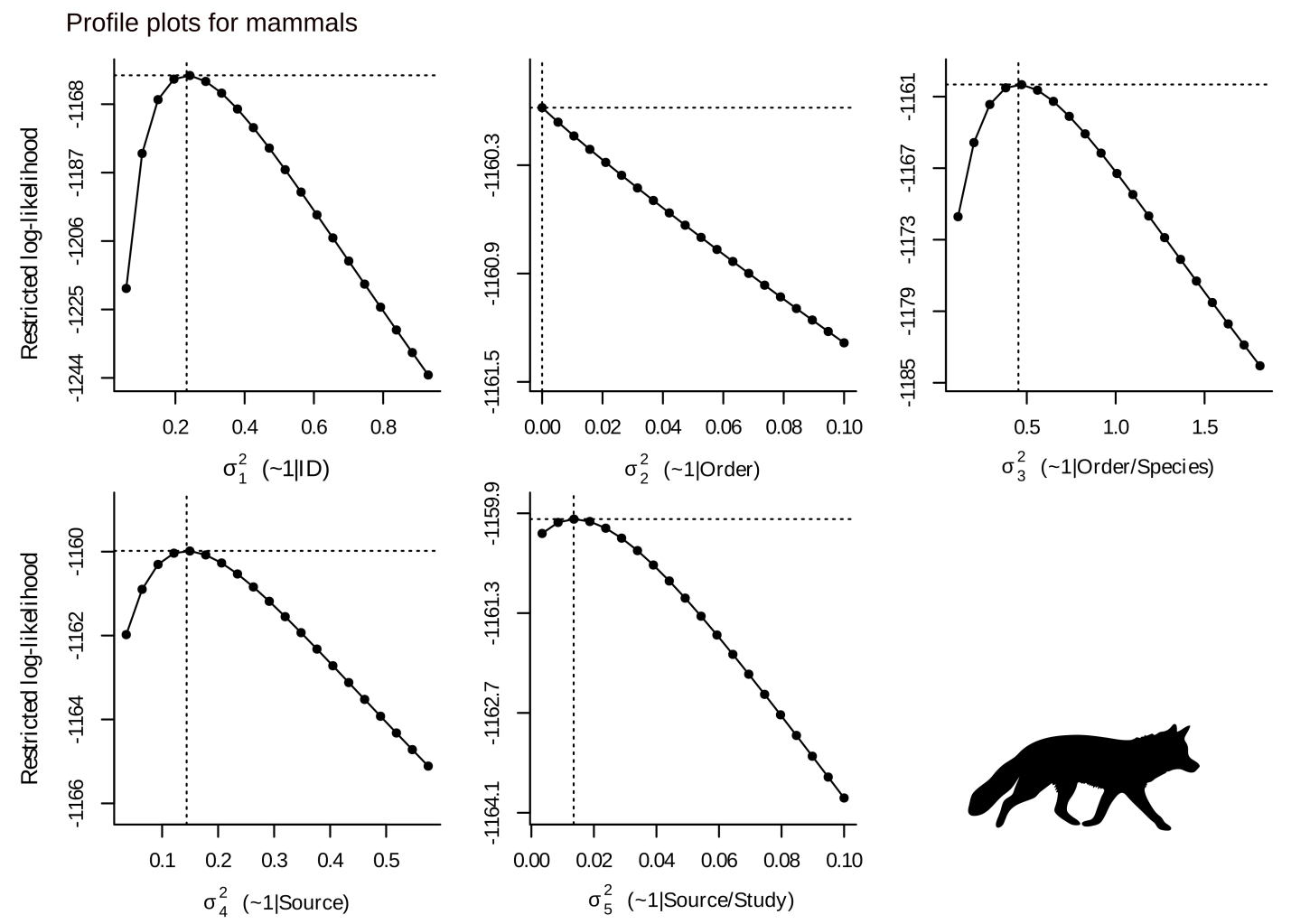


Figure S4: Profile likelihood plots of the variance components in the final model for mammals. σ^2^_1_: between-effect size variability, σ^2^_2_: between-order variability, σ^2^_3_: between-species variability, σ^2^_4_: between-sources variability, σ^2^_5_: between-study variability


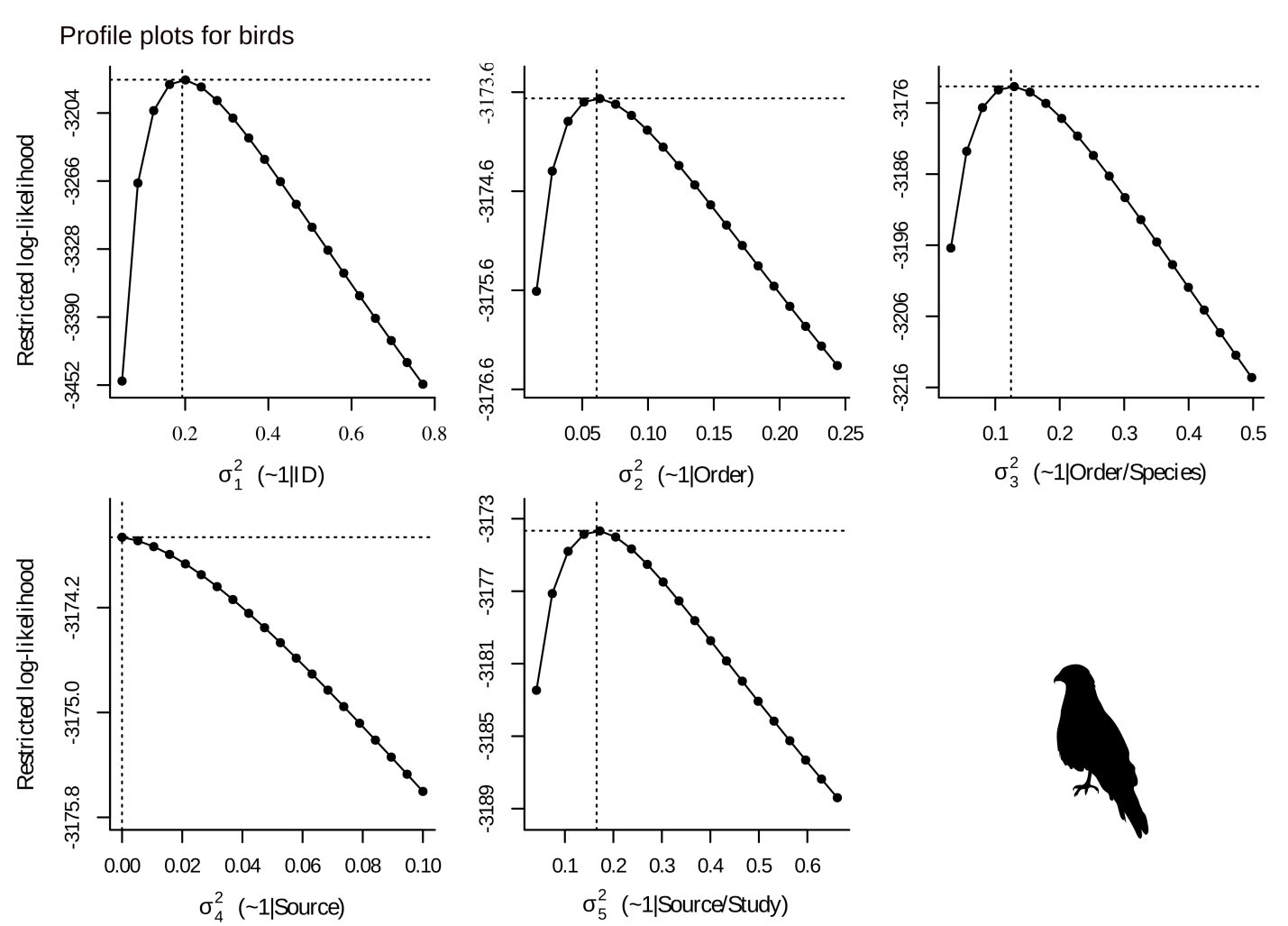


Figure S5: Profile likelihood plots of the variance components in the final model for birds. σ^2^_1_: between-effect size variability, σ^2^_2_: between-order variability, σ^2^_3_: between-species variability, σ^2^_4_: between-sources variability, σ^2^_5_: between-study variability


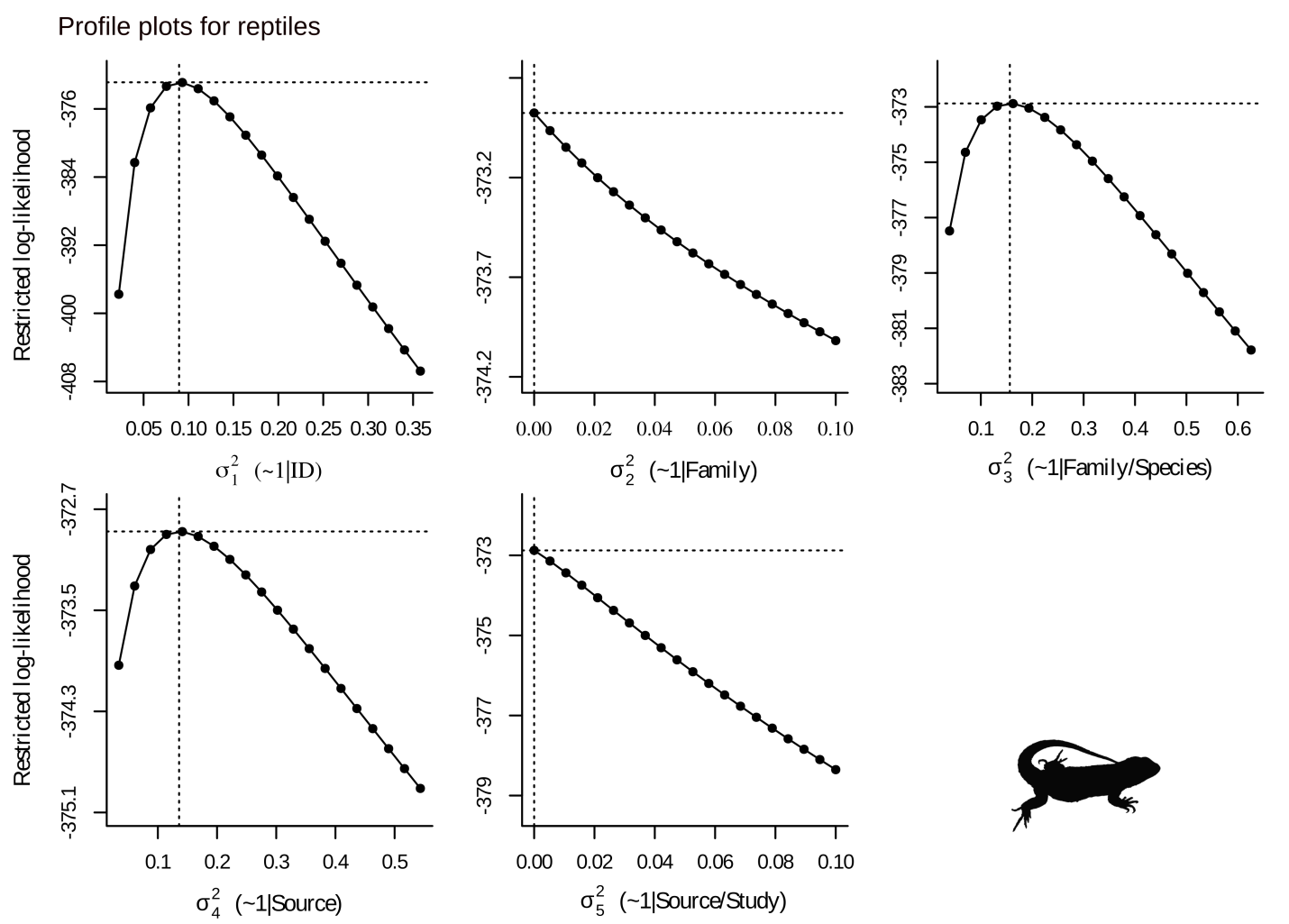


Figure S6: Profile likelihood plots of the variance components in the final model for reptiles. σ^2^_1_: between-effect size variability, σ^2^_2_: between-family variability, σ^2^_3_: between-species variability, σ^2^_4_: between-sources variability, σ^2^_5_: between-study variability


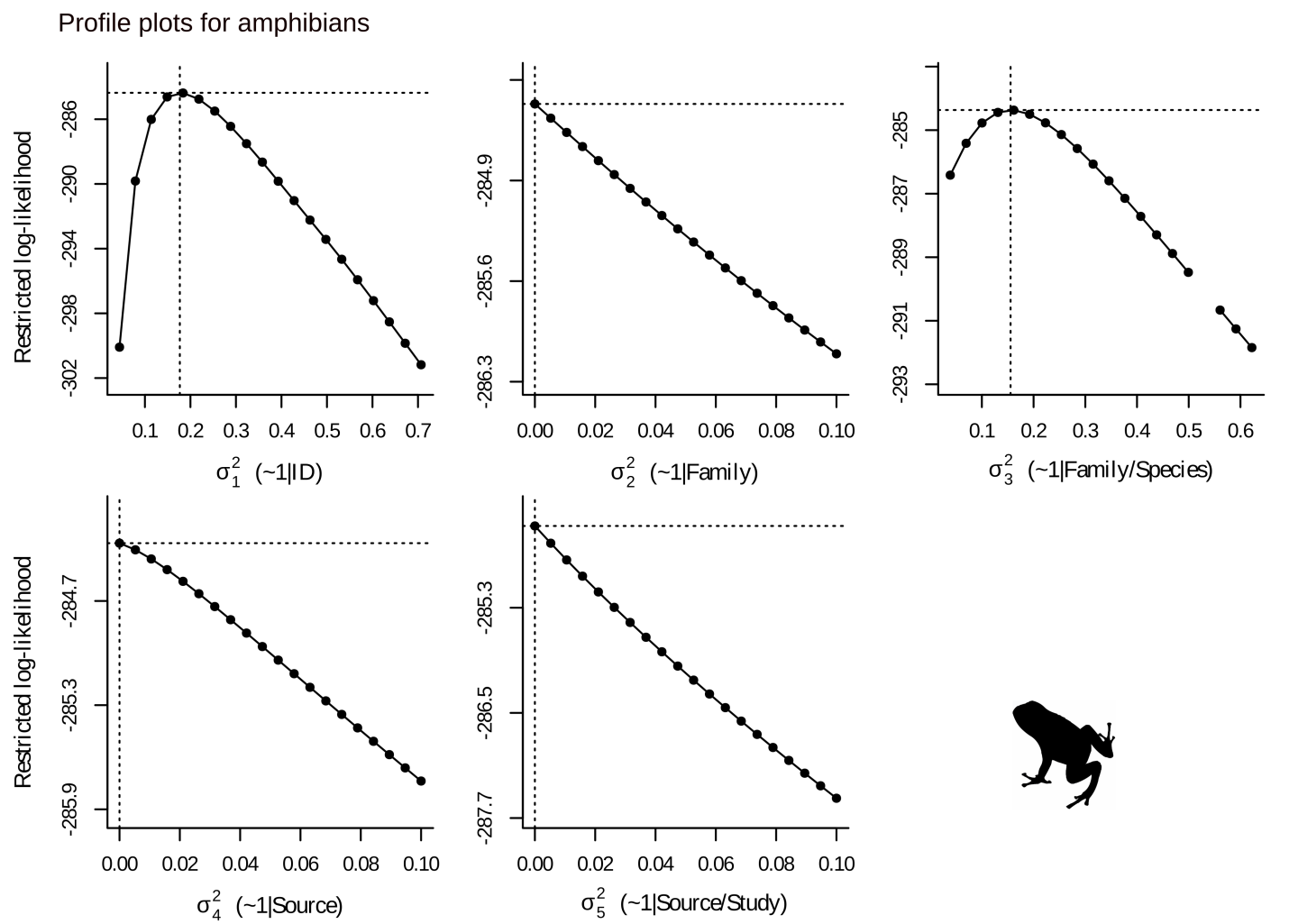


Figure S7: Profile likelihood plots of the variance components in the final model for amphibians. σ^2^_1_: between-effect size variability, σ^2^_2_: between-family variability, σ^2^_3_: between-species variability, σ^2^_4_: between-sources variability, σ^2^_5_: between-study variability


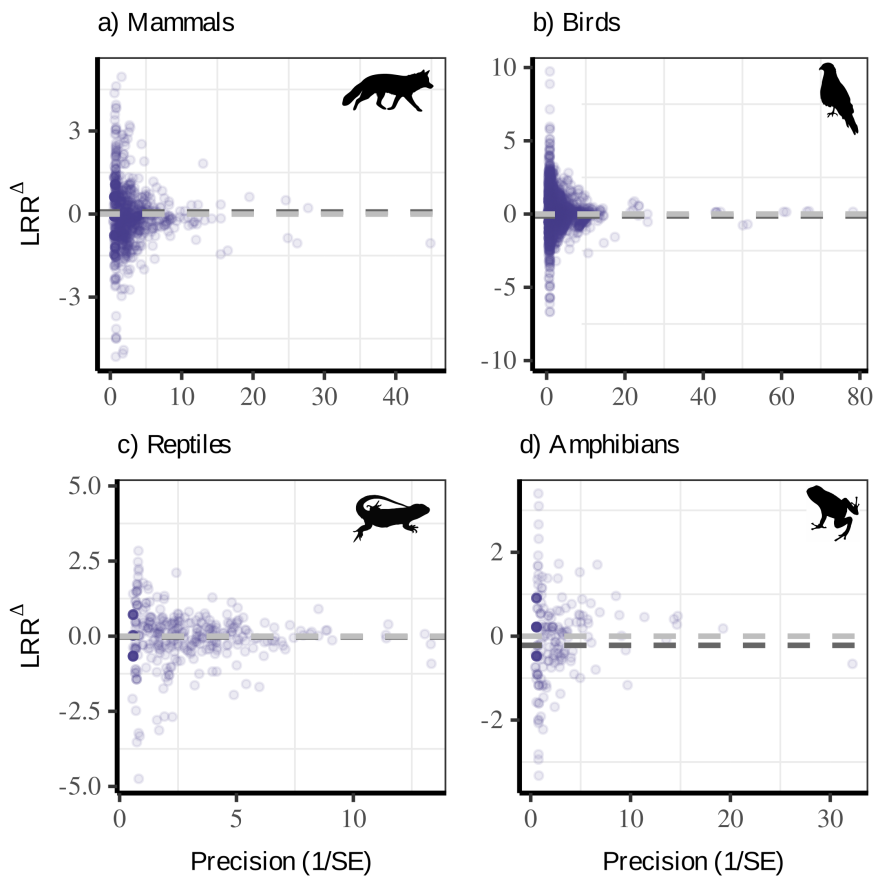


Figure S8: Funnel plots of the meta-analytic residuals for a) mammals, b) birds, d) reptiles and d) amphibians. Dashed lines indicate residual LRR^∆^ = 0 (zero line, light gray) and weighted mean effect size (dark gray). Egger test for mammals: intercept = -0.03 (95% CI: -0.27, 0.22, p=0.81). Egger test for birds: intercept = -0.-7 (95% CI: -0.27, 0.14, p=0.51). Egger test for reptiles: intercept = -0.03 (95% CI: -0.25, 0.19, p=0.82). Egger test for amphibians: intercept = -0.05 (95% CI: -0.21, 0.29, p=0.73).


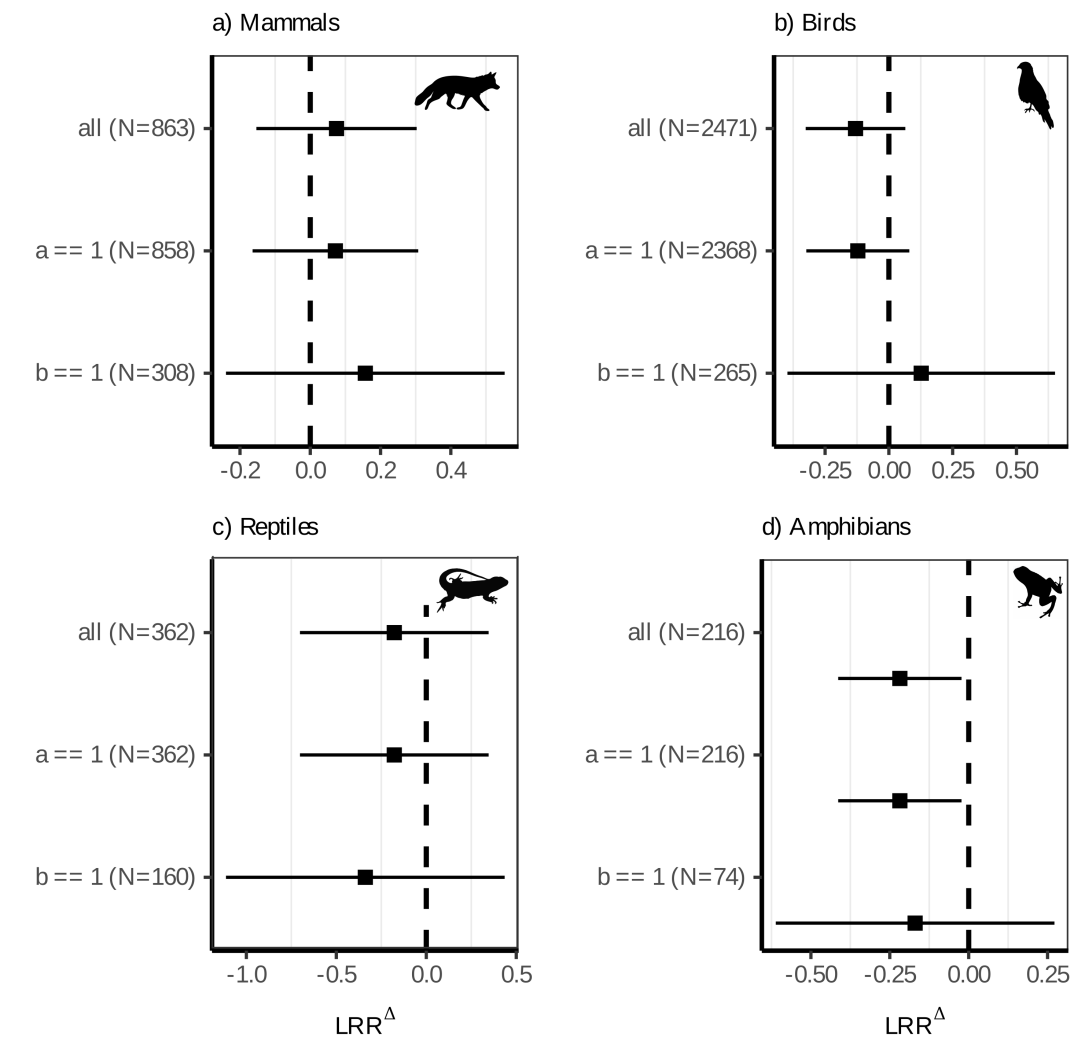
-

Figure S9: Forest plots for a) mammals, b) birds, c) reptiles and d) amphibians showing the robustness of the mean weighted effect sizes to exclusion of studies with different quality levels. all: all studies included, a == 1: excluding studies reporting abundances aggregated over 2 or more species or on genus level and b == 1: excluding studies for which the control site was not explicitly defined as undisturbed or at distances from infrastructure larger than the species’ home range. Number of retained effect sizes is indicated between brackets for each quality level (N). Dashed line indicates LRR^∆^ = 0.


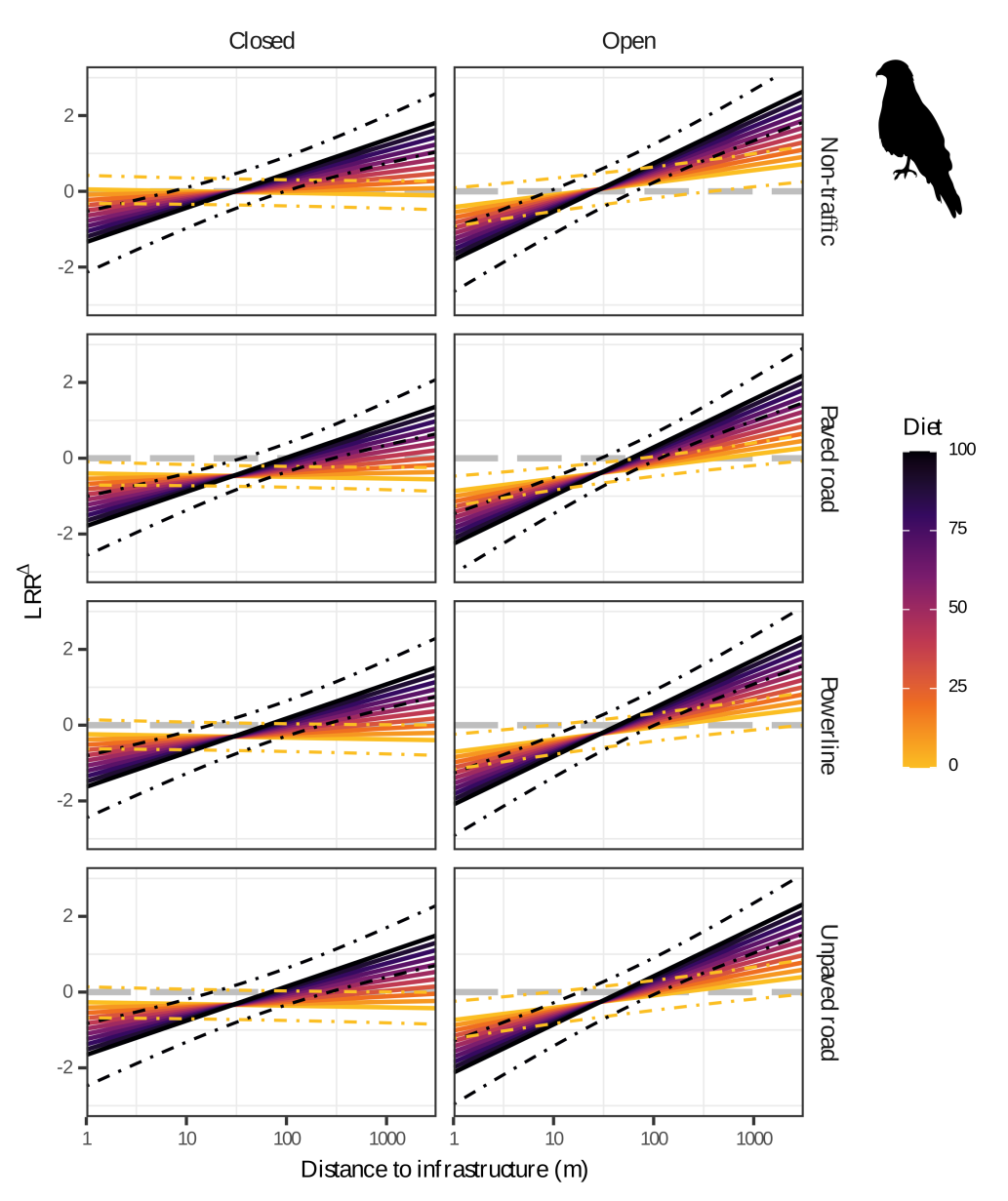


Figure S10: Change in species abundance (LRR^∆^) as a function of distance to infrastructure and diet (% of diet consisting of vertebrates or scavenging, indicated by color) for birds in closed (left panel) and open (right panel) habitats for all four infrastructure types (rows, See Table 2 for model information). Dashed lines represent the 95% confidence interval for 0 and 100% of diet consisting of vertebrates or scavenging. LRR^∆^<0 indicates abundance decline, LRR^∆^>0 indicates abundance increase and LRR^∆^=0 indicates no change (dashed grey line).


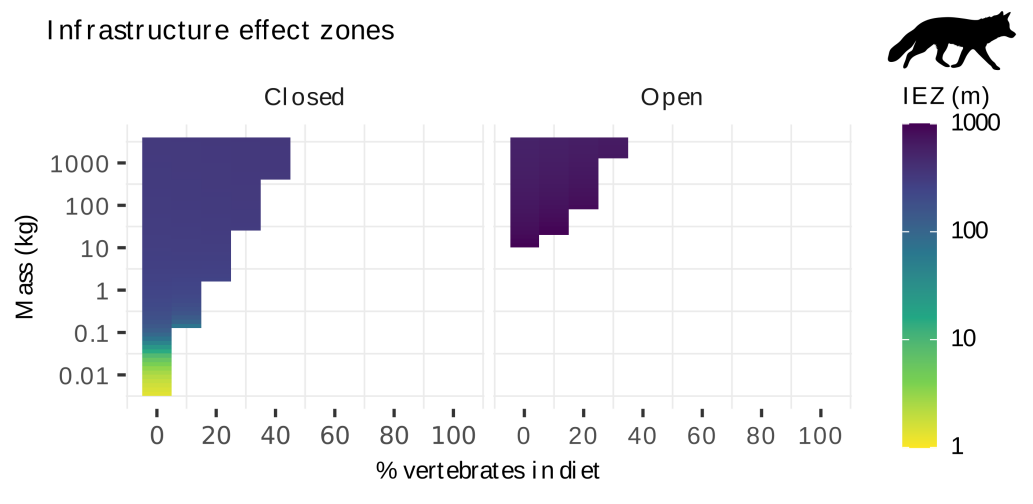


Figure S11: Estimated infrastructure effect zone (IEZ, m) for mammals as a function of body mass and diet (% of diet from vertebrates or scavenging) in closed (left panel) and open (right panel) habitats. Areas in white represent combinations of species traits and habitat type with positive responses to infrastructure.

## Supplementary Tables

Table S1: Number of effect sizes for mammals split to habitat and infrastructure type. Note that non-traffic infrastructure and power lines are combined into a single ‘non-traffic’ category in the analysis.

|  | **Paved roads** | **Unpaved roads** | **Non-traffic** | **Power line** | ***Total*** |
| --- | --- | --- | --- | --- | --- |
| **Open** | 182 | 45 | - | 7 | *234* |
| **Closed** | 291 | 180 | 65 | 93 | *629* |
| ***Total*** | *473* | *125* | *65* | *100* | *863* |

Table S2: Number of effect sizes for birds split to habitat and infrastructure type.

|  | **Paved roads** | **Unpaved roads** | **Non-traffic** | **Power line** | ***Total*** |
| --- | --- | --- | --- | --- | --- |
| **Open** | 562 | 115 | - | 92 | *769* |
| **Closed** | 679 | 43 | 227 | 753 | *1702* |
| ***Total*** | *1241* | *158* | *227* | *845* | *2471* |

Table S3: Number of effect sizes for reptiles split to habitat and infrastructure type. Note that non-traffic infrastructure and power lines are combined into a single ‘non-traffic’ category in the analysis.

|  | **Paved roads** | **Unpaved roads** | **Non-traffic** | **Power line** | ***Total*** |
| --- | --- | --- | --- | --- | --- |
| **Open** | 180 | 7 | - | - | *187* |
| **Closed** | 95 | 37 | 35 | 8 | *175* |
| ***Total*** | *275* | *44* | *35* | *8* | *362* |

Table S4: Number of effect sizes for amphibians split to habitat and infrastructure type. Note that non-traffic infrastructure and power lines are combined into a single ‘non-traffic’ category in the analysis.

|  | **Paved roads** | **Unpaved roads** | **Non-traffic** | **Power line** | ***Total*** |
| --- | --- | --- | --- | --- | --- |
| **Open** | - | - | - | - | ***-*** |
| **Closed** | 99 | 91 | 19 | 7 | *216* |
| ***Total*** | *99* | *91* | *19* | *7* | *216* |

Table S5: Parameter estimates and 95% confidence intervals and results of Omnibus Q-test (Q_M_) of random-effects meta-analysis and distance only mixed-effects meta-analysis for each taxon. k indicates the number of effect sizes included in the analysis. Asterisks indicate significance level for Q_M_: *** indicates p-value < 0.001, ** indicates p-value < 0.01, * indicates p-value < 0.05, n.s. indicates p-value > 0.05.

|  | **Mammals (*k=863)*** | | | | **Birds *(k=2471)*** | | | | **Reptiles *(k=362)*** | | | | **Amphibians *(k=216)*** | | | |
| --- | --- | --- | --- | --- | --- | --- | --- | --- | --- | --- | --- | --- | --- | --- | --- | --- |
|  | *Mean effect* | *95% CI* | *AIC* | *Q_M_* | *Mean effect* | *95% CI* | *AIC* | *Q_M_* | *Mean effect* | *95% CI* | *AIC* | *Q_M_* | *Mean effect* | *95% CI* | *AIC* | *Q_M_* |
| LRR^∆^ ~ 1 | 0.07 | -0.15, 0.30 | 2425 | ^-^ | -0.13 | -0.33, 0.06 | 6461 | - | -0.03 | -0.22, 0.17 | 827 | - | -0.22 | -0.41, -0.02 | 594 |  |
| LRR^∆^ ~ 1  + log(D) | 0.14  -0.05 | -0.10, 0.39  -0.13, 0.03 | 2426 | 1.6^n.s^ | -0.32  0.10 | -0.56, -0.08  0.03, 0.17 | 6456 | 6.9^**^ | -0.45  0.35 | -0.75, -0.16  0.25, 0.44 | 789 | 47.5^***^ | -0.51  0.24 | -0.85, -0.18  0.02, 0.46 | 592 | 4.6^*^ |
| LRR^∆^ ~ 1  + log(D)  + log(D)^2^ | 0.21  -0.28  0.09 | -0.05, 0.47  -0.51, -0.06  0.01, 0.17 | 2423 | 6.1^*^ | 0.10  -0.39  0.12 | -0.30, 0.50  -0.76, -0.01  0.03, 0.22 | 6452 | 13.5^**^ | -0.22  -0.16  0.17 | -0.55, 0.12  -0.54, 0.22  0.05, 0.30 | 784 | 57.3^***^ | -0.74  0.91  -0.32 | -1.15, -0.33  0.21, 1.62  -0.63, 0.00 | 590 | 8.7^*^ |

Table S6: Parameter estimates and 95% confidence intervals, number of effect sizes (k) and results of Cochran’s Q-test (QE) of random-effects meta-analysis based on the full dataset (all LRR^∆^) and on a subset of the data for which Geary’s diagnostic ≥ 3 (selected LRR^∆^). Asterisks indicate significance level for QE: *** indicates p-value < 0.001, ** indicates p-value < 0.01, * indicates p-value < 0.05, n.s. indicates p-value > 0.05.

|  | **Mammals** | | | | **Birds** | | | | **Reptiles** | | | | **Amphibians** | | | |
| --- | --- | --- | --- | --- | --- | --- | --- | --- | --- | --- | --- | --- | --- | --- | --- | --- |
|  | *Mean effect* | *95% CI* | *k* | *Q_E_* | *Mean effect* | *95% CI* | *k* | *Q_E_* | *Mean effect* | *95% CI* | *k* | *Q_E_* | *Mean effect* | *95% CI* | *k* | *Q_E_* |
| All LRR^∆^ | 0.07 | -0.15, 0.30 | 863 | 8410^***^ | -0.13 | -0.33, 0.06 | 2471 | 26890^***^ | -0.03 | -0.22, 0.17 | 362 | 1455^***^ | -0.22 | -0.41, -0.02 | 216 | 1407^***^ |
| Selected  LRR^∆^ | -0.04 | -0.25, 0.17 | 279 | 6646^***^ | -0.02 | -0.22, 0.17 | 943 | 15242^***^ | -0.20 | -0.50, 0.10 | 140 | 1052^***^ | -0.17 | -0.38, 0.05 | 63 | 1213^***^ |

Table S7: Results of random-effects meta-analysis under different SD imputation methods. Numbers between brackets indicate the percentage of effect sizes with imputed SDs.

|  | **Mammals (22%)** | | **Birds (63%)** | | **Reptiles (26%)** | | **Amphibians (54%)** | |
| --- | --- | --- | --- | --- | --- | --- | --- | --- |
|  | *Mean effect* | *95% CI* | *Mean effect* | *95% CI* | *Mean effect* | *95% CI* | *Mean effect* | *95% CI* |
| No imputation | 0.09 | -0.16, 0.34 | -0.01 | -0.29, 0.28 | -0.13 | -0.44, 0.17 | -0.22 | -0.58, -0.07 |
| Poisson | 0.07 | -0.15, 0.30 | -0.13 | -0.33, 0.06 | -0.03 | -0.22, 0.17 | -0.22 | -0.41, -0.02 |
| Bracken | 0.06 | -0.17, 0.29 | -0.22 | -0.43, 0.00 | -0.00 | -0.20, 0.19 | -0.33 | -0.59, -0.08 |
| HotDeckNN (median) | 0.06 | -0.17, 0.29 | -0.20 | -0.42, 0.02 | -0.01 | -0.21, 0.20 | -0.25 | -0.48, -0.02 |
| HotDeckNN (mean) | 0.06 | -0.17, 0.29 | -0.20 | -0.42, 0.02 | -0.01 | -0.21, 0.20 | -0.24 | -0.47, -0.01 |

Table S8: Results of mixed-effects meta-regression model selection for mammals with Akaike information criterium (AICc) and difference from the lowest AICc (∆AICc). Only the 10 models with the lowest AICc and those with only one moderator are shown for simplicity.

| **Fixed effects** | **AICc** | **∆AICc** |
| --- | --- | --- |
| ~logD * Diet + logD * Habitat + logD * logBM + logD^2^ * logBM | 2374.6 | - |
| ~logD * Diet + logD * Habitat + logD^2^ * Habitat + logD * logBM + logD^2^ * logBM | 2375.4 | 0.8 |
| ~logD * Diet + logD^2^ * Diet + logD * Habitat + logD * logBM + logD^2^ * logBM | 2376.7 | 2.1 |
| ~logD * Diet + logD^2^ * Diet + logD * Habitat + logD^2^ * Habitat + logD * logBM + logD^2^ * logBM | 2377.5 | 2.9 |
| ~logD * Diet + logD * logBM + logD^2^ * logBM + logD * Habitat + InfraType | 2378.1 | 3.5 |
| ~logD * Diet + logD * logBM + logD^2^ * logBM + logD * Habitat + logD^2^ * Habitat + InfraType | 2378.9 | 4.3 |
| ~logD * Diet + logD * logBM + logD^2^ * logBM | 2380.2 | 5.6 |
| ~logD * Diet + logD^2^ * Diet + logD * logBM + logD^2^ * logBM + logD * Habitat + InfraType | 2380.2 | 5.6 |
| ~logD * Diet + logD^2^ * Diet + logD * logBM + logD^2^ * logBM + logD * Habitat + logD^2^ * Habitat + InfraType | 2381.1 | 6.4 |
| ~logD * Diet + logD * logBM + logD^2^ * logBM + Habitat | 2382.0 | 7.4 |
| ~Diet | 2415.8 | 41.2 |
| ~logD + logD^2^ | 2423.3 | 48.7 |
| ~1 | 2425.3 | 50.7 |
| ~logD | 2425.7 | 51.1 |
| ~logBM | 2426.4 | 51.8 |
| ~Habitat | 2426.6 | 51.9 |
| ~InfraType | 2429.2 | 54.5 |

Table S9: Results of mixed-effects meta-regression model selection for birds with Akaike information criterium (AICc) and difference from the lowest AICc (∆AICc). Only the 10 models with the lowest AICc and those with only one moderator are shown for simplicity.

| **Fixed effects** | **AICc** | **∆AICc** |
| --- | --- | --- |
| ~logD * Diet + logD * Habitat + InfraType | 6420.2 | 0.0 |
| ~logD * Diet + logD * logBM + logD * Habitat + InfraType | 6420.3 | 0.2 |
| ~logD * Diet + logD * Habitat | 6420.5 | 0.3 |
| ~logD * Diet + logD * Habitat + logD * logBM | 6420.9 | 0.7 |
| ~logD * Diet + logD^2^ + logD * Habitat + InfraType | 6421.7 | 1.5 |
| ~logD * Diet + logBM + logD * Habitat + InfraType | 6422.0 | 1.8 |
| ~logD * Diet + logD * logBM + logD * Habitat + InfraType + logD^2^ | 6422.1 | 2.0 |
| ~logD * Diet + logD^2^ + logD * Habitat | 6422.2 | 2.0 |
| ~logD * Diet + logD * Habitat + logBM | 6422.3 | 2.1 |
| ~logD * Diet + logD * logBM + logD^2^ * logBM + logD * Habitat + InfraType | 6422.6 | 2.5 |
| ~logD + logD^2^ | 6451.9 | 31.8 |
| ~logD | 6456.3 | 36.1 |
| ~Habitat | 6460.4 | 40.2 |
| ~1 | 6461.2 | 41.0 |
| ~logBM | 6461.7 | 41.6 |
| ~Diet | 6461.9 | 41.7 |
| ~InfraType | 6466.2 | 46.0 |

Table S10: Results of mixed-effects meta-regression model selection for reptiles with Akaike information criterium (AICc) and difference from the lowest AICc (∆AICc). Only the 10 models with the lowest AICc and those with only one moderator are shown for simplicity.

| **Fixed effects** | **AICc** | **∆AICc** |
| --- | --- | --- |
| ~logD * Habitat + logD^2^ | 781.6 | 0.0 |
| ~logD * Habitat + logD^2^ + logBM | 781.6 | 0.0 |
| ~logD * logBM + logD^2^ + logD * Habitat | 782.3 | 0.7 |
| ~logD * logBM + logD^2^ * logBM + logD * Habitat | 782.8 | 1.2 |
| ~logD * Habitat + logD^2^ * Habitat | 783.0 | 1.4 |
| ~logD * Habitat + logD^2^ * Habitat + logBM | 783.2 | 1.6 |
| ~logD + logD^2^ + InfraType | 783.4 | 1.8 |
| ~logD + logD^2^ + logBM + InfraType | 783.8 | 2.2 |
| ~logD * logBM + logD * Habitat + logD^2^ * Habitat | 783.9 | 2.4 |
| ~logD * Habitat + logBM | 784.4 | 2.8 |
| ~logD + logD^2^ | 784.5 | 2.9 |
| ~logD | 789.4 | 7.9 |
| ~logBM | 826.5 | 44.9 |
| ~1 | 826.8 | 45.2 |
| ~Habitat | 828.9 | 47.3 |
| ~InfraType | 829.5 | 47.9 |

Table S11: Results of mixed-effects meta-regression model selection for amphibians with Akaike information criterium (AICc) and difference from the lowest AICc (∆AICc).

| **Fixed effects** | **AICc** | **∆AICc** |
| --- | --- | --- |
| ~logD + logD^2^ | 590.4 | 0.0 |
| ~logD + logD^2^ + logBM | 590.7 | 0.3 |
| ~logD * logBM + logD^2^ | 591.7 | 1.4 |
| ~logD | 591.9 | 1.5 |
| ~logD * logBM + logD^2^ * logBM | 592.0 | 1.6 |
| ~logD + logBM | 592.5 | 2.2 |
| ~1 | 594.2 | 3.8 |
| ~logD * logBM | 594.2 | 3.8 |
| ~logD + logD^2^ + InfraType | 595.0 | 4.7 |
| ~logBM | 595.3 | 4.9 |
| ~logD + logD^2^ + logBM + InfraType | 595.3 | 5.0 |
| ~logD + InfraType | 596.0 | 5.6 |
| ~InfraType | 596.0 | 5.6 |
| ~logD + logBM + InfraType | 596.2 | 5.8 |
| ~logD * logBM + logD^2^ + InfraType | 596.6 | 6.3 |
| ~logD * logBM + logD^2^ * logBM + InfraType | 596.8 | 6.5 |
| ~logD * logBM + InfraType | 597.6 | 7.3 |

Table S12: Estimates of variance components (σ_1_^2^: observation-level variability, σ_2_^2^: order or family - level variability, σ_3_^2^: species-level variability, σ_4_^2^: source–level variability, σ_5_^2^: study-level variability) of the final model selected based on the AICc for mammals, birds, reptiles and amphibians.

|  | **Mammals** | **Birds** | **Reptiles** | **Amphibians** |
| --- | --- | --- | --- | --- |
| σ_1_^2^ (residual) | 0.2325 | 0.1940 | 0.0902 | 0.1767 |
| σ_2_^2^ (order/family) | 0.0000 | 0.0505 | 0.0000 | 0.0000 |
| σ_3_^2^ (species) | 0.4529 | 0.1242 | 0.1517 | 0.1555 |
| σ_4_^2^ (source) | 0.1437 | 0.0000 | 0.1400 | 0.0000 |
| σ_5_^2^ (study) | 0.0138 | 0.1702 | 0.0000 | 0.0000 |

## Data sources

Ascensao, F., Clevenger, A. P., Grilo, C., Filipe, J., & Santos-Reis, M. (2012). Highway verges as habitat providers for small mammals in agrosilvopastoral environments. *Biodiversity and Conservation*, 21(14), 3681-3697.

Aldridge, C. A. (2017). *The effect of road crossings on stream-associated salamanders within Holly Springs National Forest* (Document No. 1274) [MSc thesis, University of Mississippi]. Electronic Theses and Dissertations.

Astudillo, P. X., Samaniego, G. M., Machado, P. J., Aguilar, J. M., Tinoco, B. A., Graham, C. H., ... & Farwig, N. (2014). The impact of roads on the avifauna of páramo grasslands in Cajas National Park, Ecuador. *Studies on neotropical fauna and environment*, 49(3), 204-212.

Bager, A., & da Rosa, C. A. (2012). Impacts of the BR-392 highway on bird communities in extreme southern Brazil. *Revista Brasileira de Ornitologia*, 20(1), 30-39.

Ballasus, H., & Sossinka, R. (1997). The impact of power lines on field selection and grazing intensity of wintering White-fronted-and Bean Geese *Anser albifrons, A. fabalis*. *Journal Fur Ornithologie*, 138(2), 215-228.

Barrows, C. W., Allen, M. F., & Rotenberry, J. T. (2006). Boundary processes between a desert sand dune community and an encroaching suburban landscape. *Biological Conservation*, 131(4), 486-494.

Barton, D. C., & Holmes, A. L. (2007). Off-highway vehicle trail impacts on breeding songbirds in northeastern California. *The Journal of wildlife management*, 71(5), 1617-1620.

Bautista, L. M., García, J. T., Calmaestra, R. G., Palacín, C., Martín, C. A., Morales, M. B., ... & Viñuela, J. (2004). Effect of weekend road traffic on the use of space by raptors. *Conservation Biology*, 18(3), 726-732.

Bayne, E., Leston, L., Mahon, C. L., Sólymos, P., Machtans, C., Lankau, H., ... & Schmiegelow, F. K. (2016). Boreal bird abundance estimates within different energy sector disturbances vary with point count radius. *The Condor: Ornithological Applications*, 118(2), 376-390.

Bissonette, J., & Rosa, S. (2009). Road Zone Effects in Small-Mammal Communities. *Ecology and Society*, 14(1), 27

Boarman, W. I., & Sazaki, M. (2006). A highway's road-effect zone for desert tortoises *(Gopherus agassizii)*. *Journal of Arid Environments*, 65(1), 94-101.

Braga, C. A. D. C., Prevedello, J. A., & Pires, M. R. S. (2015). Effects of cornfields on small mammal communities: a test in the Atlantic Forest hotspot. *Journal of Mammalogy*, 96(5), 938-945.

Bramble, W. C., Byrnes, W. R., & Schuler, M. D. (1984). The bird population of a transmission right-of-way maintained by herbicides. *Journal of Arboriculture*, 10(1), 13-20.

Brearley, G., Bradley, A., Bell, S., & McAlpine, C. (2010). Influence of contrasting urban edges on the abundance of arboreal mammals: a study of squirrel gliders (*Petaurus norfolcensis*) in southeast Queensland, Australia. *Biological Conservation*, 143(1), 60-71.

Brehme, C. S. (2003). *Responses of Small Terrestrial Vertebrates to Roads in Coastal Sage Scrub Ecosystem* [MSc Thesis, San Diego State University]. Retrieved from https://escholarship.org/uc/item/94h8j1g3

Cameron, R. D., Reed, D. J., Dau, J. R., & Smith, W. T. (1992). Redistribution of calving caribou in response to oil field development on the Arctic Slope of Alaska. *Arctic*, 45(4), 338-342.

Canaday, C. (1996). Loss of insectivorous birds along a gradient of human impact in Amazonia. *Biological Conservation*, 77(1), 63-77.

Cappa, F. M., Giannoni, S. M., Ontiveros, Y., & Borghi, C. E. Direct and indirect effects of roads on activity patterns of the largest South American artiodactyl (*Lama guanicoe*) in a hyper‑arid landscape. *Mammalian biology*, 100, 453-461

Cappa, F. M., Borghi, C. E., & Giannoni, S. M. (2019). How Roads Affect the Spatial Use of the Guanaco in a South American Protected Area: Human Connectivity vs Animal Welfare. *Diversity*, 11(7), 110.

Carthew, S. M., Garrett, L. A., & Ruykys, L. (2013). Roadside vegetation can provide valuable habitat for small, terrestrial fauna in South Australia. *Biodiversity and conservation,* 22(3), 737-754.

Carthew, S. M., Jones, K. M., & Lawes, M. (2013). Responses of small vertebrates to linear clearings in a South Australian woodland. *Ecological research*, 28(6), 1003-1010.

Chen, W., Zhong, J., Carson, W. P., Tang, Z., Xie, Z., Sun, S., & Zhou, Y. (2019). Proximity to roads disrupts rodents’ contributions to seed dispersal services and subsequent recruitment dynamics. *Journal of Ecology*, 107(6), 2623-2634.

Chen, W., Xie, Z., & Zhou, Y. (2019). Proximity to roads reduces acorn dispersal effectiveness by rodents: Implication for forest regeneration and management. *Forest Ecology and Management*, 433, 625-632.

Chiarello, A., Srbek-Araujo, A., Del-Duque Jr, H., de Coelho, E., & Rocha, C. (2010). Abundance of tegu lizards (*Tupinambis merianae*) in a remnant of the Brazilian Atlantic forest. *Amphibia-Reptilia*, 31(4), 563-570.

Clark, W. D., & Karr, J. R. (1979). Effects of highways on Red-winged Blackbird and Horned Lark populations. *The Wilson Bulletin*, 91(1), 143-145.

Clarke, D. J., & White, J. G. (2008). Recolonisation of powerline corridor vegetation by small mammals: timing and the influence of vegetation management. *Landscape and urban planning*, 87(2), 108-116.

Clarke, R. T., Liley, D., Sharp, J. M., & Green, R. E. (2013). Building development and roads: Implications for the distribution of stone curlews across the Brecks. *PloS one*, 8(8).

Darling, A. F., Leston, L., & Bayne, E. M. (2019). Small-mammal abundance differs between pipelines, edges, and interior boreal forest habitat. *Canadian Journal of Zoology*, 97(10), 880-894.

Delgado, J. D., Arévalo, J. R., & Fernández-Palacios, J. M. (2004). Consecuencias de la fragmentación viaria: efectos de borde de las carreteras en la laurisilva y el pinar de Tenerife. *Ecología Insular/Island Ecology.* Asociación Española de Ecología Terrestre (AEET)-Cabildo Insular de la Palma, 181-225.

Delgado, J. D., Arévalo, J. R., & Fernández-Palacios, J. M. (2008). Bird communities in two oceanic island forests fragmented by roads on Tenerife, Canary Islands. *Ostrich-Journal of African Ornithology*, 79(2), 219-226.

Develey, P. F., & Stouffer, P. C. (2001). Effects of roads on movements by understory birds in mixed‐species flocks in central Amazonian Brazil. *Conservation biology*, 15(5), 1416-1422.

Dorland, A., Rytwinski, T., & Fahrig, L. (2014). Do roads reduce painted turtle (*Chrysemys picta*) populations?. *PloS one*, 9(5), e98414.

Fleming, W. D. (2001). Effects of pipeline rights-of-way on forest birds in the boreal forest of Alberta.[MSc thesis, University of Alberta], ProQuest

Floyd, K. W. (2015). *An assessment of the role of roads in population demography and genetic structuring in two species of lizards in the northern Chihuahuan Desert* (ProQuest No. 3724920). [Doctoral dissertation, The University of Texas at El Paso]. ProQuest

García, J. D. D., Arévalo, J. R., & Fernández-Palacios, J. M. (2007). Road edge effect on the abundance of the lizard *Gallotia gallot*i (Sauria: Lacertidae) in two Canary Islands forests. *Biodiversity and Conservation*, 16(10), 2949-2963.

Gill, J. A., Sutherland, W. J., & Watkinson, A. R. (1996). A method to quantify the effects of human disturbance on animal populations. *Journal of applied Ecology*, 33(4), 786-792.

Goldingay, R. L., & Whelan, R. J. (1997). Powerline easements: do they promote edge effects in eucalypt forest for small mammals?. *Wildlife Research*, 24(6), 737-744.

Goodwin, S. E., & Shriver, W. G. (2011). Effects of traffic noise on occupancy patterns of forest birds. *Conservation Biology*, 25(2), 406-411.

Goosem, M., & Marsh, H. (1997). Fragmentation of a small-mammal community by a powerline corridor through tropical rainforest. *Wildlife Research*, 24(5), 613-629.

Goosem, M. (2000). Effects of tropical rainforest roads on small mammals: edge changes in community composition. *Wildlife Research*, 27(2), 151-163.

Goosem, M. (2002). Effects of tropical rainforest roads on small mammals: fragmentation, edge effects and traffic disturbance. *Wildlife Research*, 29(3), 277-289.

Herrera-Montes, M. I., & Aide, T. M. (2011). Impacts of traffic noise on anuran and bird communities. *Urban Ecosystems*, 14(3), 415-427.

Hoskin, C. J., & Goosem, M. W. (2010). Road impacts on abundance, call traits, and body size of rainforest frogs in northeast Australia. *Ecology and society,* 15(3), 15.

Hughson, D. L., & Darby, N. (2013). Desert tortoise road mortality in Mojave National Preserve, California. *California Fish and Gam*e, 99, 222-232.

Hunkapiller, T. R., Ford, N. B., & Herriman, K. (2009). The effects of all-terrain vehicle use on the herpetofauna of an east Texas floodplain. *Texas Journal of Science*, 61(1), 3-14.

Huijser, M. P., & Bergers, P. J. (2000). The effect of roads and traffic on hedgehog (*Erinaceus europaeus*) populations. *Biological conservation*, 95(1), 111-116.

Jack, J. R. (2013). Investigating the role of mortality in explaining the negative road effect on birds [MSc thesis, Carleton University].

Ji, S., Jiang, Z., Li, L., Li, C., Zhang, Y., Ren, S., ... & Chu, H. (2017). Impact of different road types on small mammals in Mt. Kalamaili Nature Reserve. *Transportation Research Part D: Transport and Environment*, 50, 223-233.

Jones, D. N., Bernede, L., Bond, A. R. F., Dexter, C., & Strong, C. L. (2016). Dust as a contributor to the road-effect zone: a case study from a minor forest road in Australia. *Australasian Journal of Environmental Management*, 23(1), 67-80.

King, C. M., Innes, J. G., Flux, M., & Kimberley, M. O. (1996). Population biology of small mammals in Pureora Forest Park: 2. The feral house mouse *(Mus musculus*). *New Zealand Journal of Ecology*, 20(2), 253-269.

Khamcha, D., Powell, L. A., & Gale, G. A. (2018). Effects of roadside edge on nest predators and nest survival of Asian tropical forest birds. *Global Ecology and Conservation*, 16, e00450.

Knight, R. L., & Kawashima, J. Y. (1993). Responses of raven and red-tailed hawk populations to linear right-of-ways. *The Journal of wildlife management*, 57(2), 266-271.

Kroodsma, R. L. (1984). Effect of edge on breeding forest bird species. *The Wilson Bulletin*, 96(3), 426-436.

Kuitunen, M., Rossi, E., & Stenroos, A. (1998). Do highways influence density of land birds?. *Environmental management*, 22(2), 297-302.

Lambertucci, S. A., Speziale, K. L., Rogers, T. E., & Morales, J. M. (2009). How do roads affect the habitat use of an assemblage of scavenging raptors?. *Biodiversity and Conservation*, 18(8), 2063-2074.

Lane, S. J., Alonso, J. C., & Martín, C. A. (2001). Habitat preferences of great bustard *Otis tarda* flocks in the arable steppes of central Spain: are potentially suitable areas unoccupied?. *Journal of applied ecology*, 38(1), 193-203.

Langenberger, K. C. (2006). *Geese protection vs. settlement: The distribution and land use of Greenland White-fronted Geese on Hvanneyri fields and their reaction to disturbances* (BSc thesis, Agricultural University of Iceland).

Laurance, W. F., Croes, B. M., Tchignoumba, L., Lahm, S. A., Alonso, A., Lee, M. E., ... & Ondzeano, C. (2006). Impacts of roads and hunting on central African rainforest mammals. *Conservation Biology*, 20(4), 1251-1261.

Lee, E. (2006). *The ecological effects of sealed roads in arid ecosystems* [Doctoral dissertation, University of New South Wales].

Letto (2013). *Living with linear features: impact of roads, recreational trails and transmission line rights-of-way on small mammals in Newfoundland* [Doctoral dissertation, Memorial University of Newfoundland].

Lituma, C. M. (2014). *Regional Assessment of the Relationships of Conservation Practices to Northern Bobwhite and Other Priority Grassland Bird Breeding Populations.*(Document No. 2706) [Doctoral dissertation, University of Tennessee]. Retrieved from https://trace.tennessee.edu/utk_graddiss/2706

Lolya, L. M. (2019). *Assessing Avian Responses to Habitat Management Along Pipeline Right-of-ways in Eastern Ohio* [Doctoral dissertation, The Ohio State University].

Luck, G. W., Possingham, H. P., & Paton, D. C. (1999). Bird responses at inherent and induced edges in the Murray Mallee, South Australia. 1. Differences in abundance and diversity. *Emu*, 99(3), 157-169.

Malcolm, J. R., & Ray, J. C. (2000). Influence of timber extraction routes on central African small-mammal communities, forest structure, and tree diversity. *Conservation Biology*, 14(6), 1623-1638.

Marsh, D. M. (2007). Edge effects of gated and ungated roads on terrestrial salamanders. The *Journal of wildlife management*, 71(2), 389-394.

Marsh, D. M., & Beckman, N. G. (2004). Effects of forest roads on the abundance and activity of terrestrial salamanders. *Ecological applications*, 14(6), 1882-1891

Maynard, R. J., Aall, N. C., Saenz, D., Hamilton, P. S., & Kwiatkowski, M. A. (2016). Road-edge effects on herpetofauna in a lowland Amazonian rainforest. *Tropical Conservation Science*, 9(1), 264-290.

McGregor, R. L., Bender, D. J., & Fahrig, L. (2008). Do small mammals avoid roads because of the traffic?. *Journal of Applied Ecology*, 45(1), 117-123.

Medinas, D., Ribeiro, V., Marques, J. T., Silva, B., Barbosa, A. M., Rebelo, H., & Mira, A. (2019). Road effects on bat activity depend on surrounding habitat type. *Science of the Total Environment*, 660, 340-347.

Meunier, F. D., Corbin, J., Verheyden, C., & Jouventin, P. (1999). Effects of landscape type and extensive management on use of motorway roadsides by small mammals. *Canadian Journal of Zoology*, 77(1), 108-117.

Meunier, F. D., Verheyden, C., & Jouventin, P. (1999). Bird communities of highway verges: influence of adjacent habitat and roadside management. *Acta Oecologica*, 20(1), 1-13.

Meunier, F. D., Verheyden, C., & Jouventin, P. (2000). Use of roadsides by diurnal raptors in agricultural landscapes. *Biological Conservation*, 92(3), 291-298.

Mohd-Azlan, J. (2018). Distribution, relative abundance and occupancy of selected mammals along paved road in Kubah National Park, Sarawak, Borneo. Nature Conservation Research. *Заповедная наука*, 3(2) 36-46.

Nafus, M. G., Tuberville, T. D., Buhlmann, K. A., & Todd, B. D. (2013). Relative abundance and demographic structure of Agassiz’s desert tortoise (*Gopherus agassizi*i) along roads of varying size and traffic volume. *Biological Conservation*, 162, 100-106.

Nellemann, C., Vistnes, I., Jordhøy, P., & Strand, O. (2001). Winter distribution of wild reindeer in relation to power lines, roads and resorts. *Biological conservation*, 101(3), 351-360.

Newmark, W. D., Boshe, J. I., Sariko, H. I., & Makumbule, G. K. (1996). Effects of a highway on large mammals in Mikumi National Park, Tanzania. *African Journal of Ecology*, 34(1), 15-31.

Niemi, G. J., & Hanowski, J. M. (1984). Effects of a transmission line on bird populations in the Red Lake Peatland, northern Minnesota. *The Auk,* 101(3), 487-498.

Noel, L. E., Parker, K. R., & Cronin, M. A. (2004). Caribou distribution near an oilfield road on Alaska's North Slope, 1978–2001. *Wildlife Society Bulletin*, 32(3), 757-771.

Ortega, Y. K., & Capen, D. E. (1999). Effects of forest roads on habitat quality for ovenbirds in a forested landscape. *The Auk*, 116(4), 937-946.

Pattison, C. A., Castley, J. G., & Catterall, C. P. (2020). Seismic linear clearings alter mammal abundance and community composition in boreal forest landscapes. *Forest Ecology and Management*, 462, 117936.

Peaden, J. M., Tuberville, T. D., Buhlmann, K. A., Nafus, M. G., & Todd, B. D. (2015). Delimiting road-effect zones for threatened species: implications for mitigation fencing. *Wildlife Research*, 42(8), 650-659.

Planillo, A., & Malo, J. E. (2013). Motorway verges: Paradise for prey species? A case study with the European rabbit. *Mammalian Biology*, 78(3), 187-192.

Planillo, A., Mata, C., Manica, A., & Malo, J. E. (2018). Carnivore abundance near motorways related to prey and roadkills. *The Journal of Wildlife Management*, 82(2), 319-327.

Polak, M., Wiącek, J., Kucharczyk, M., & Orzechowski, R. (2013). The effect of road traffic on a breeding community of woodland birds. *European Journal of Forest Research*, 132(5-6), 931-941.

Raiter, K. G., Hobbs, R. J., Possingham, H. P., Valentine, L. E., & Prober, S. M. (2018). Vehicle tracks are predator highways in intact landscapes. *Biological Conservation*, 228, 281-290.

Reijnen, R., & Foppen, R. (1994). The effects of car traffic on breeding bird populations in woodland. I. Evidence of reduced habitat quality for willow warblers (*Phylloscopus trochilus*) breeding close to a highway. *Journal of Applied ecology,* 31(1) 85-94.

Reijnen, R., Foppen, R., Braak, C. T., & Thissen, J. (1995). The effects of car traffic on breeding bird populations in woodland. III. Reduction of density in relation to the proximity of main roads. *Journal of Applied ecolog*y, 32(1) 187-202.

Rich, A. C., Dobkin, D. S., & Niles, L. J. (1994). Defining forest fragmentation by corridor width: the influence of narrow forest‐dividing corridors on forest‐nesting birds in southern New Jersey. *Conservation Biology*, 8(4), 1109-1121.

Roedenbeck, I. A., & Voser, P. (2008). Effects of roads on spatial distribution, abundance and mortality of brown hare (*Lepus europaeus*) in Switzerland. *European Journal of Wildlife Research*, 54(3), 425-437.

Rost, G. R., & Bailey, J. A. (1979). Distribution of mule deer and elk in relation to roads. The *Journal of Wildlife Management*, 43(3), 634-641.

Rotenberry, J. T., & Knick, S. T. (1995). Evaluation of bias in roadside point count surveys of passerines in shrubsteppe and grassland habitats in southwestern Idaho. In: C. J. Ralph; J. S. Sauer; S. Droege (Eds.), Monitoring bird populations by point counts. Gen. Tech. Rep. PSW-GTR-149 (pp 99-102, 149). Albany, CA: US Department of Agriculture, Forest Service, Pacific Southwest Research Station

Ruiz-Capillas, P., Mata, C., & Malo, J. E. (2013). Road verges are refuges for small mammal populations in extensively managed Mediterranean landscapes. *Biological conservation*, 158, 223-229.

Sabino-Marques, H., & Mira, A. (2011). Living on the verge: are roads a more suitable refuge for small mammals than streams in Mediterranean pastureland?. *Ecological research*, 26(2), 277-287.

Semlitsch, R. D., Ryan, T. J., Hamed, K., Chatfield, M., Drehman, B., Pekarek, N., ... & Watland, A. (2007). Salamander abundance along road edges and within abandoned logging roads in Appalachian forests. *Conservation biology*, 21(1), 159-167.

Smith, D.J., Grace, M.K., Chasez, H.R. & Noss, M.J.W. (2015). State Road 40 Pre-Construction Wildlife Movement Monitoring: Areas A, B and F. Final Report, Contract No. BDK78, TWO #501-3. Florida Department of Transportation, District Five, Deland,

Storm, J. J., & Choate, J. R. (2012). Structure and movements of a community of small mammals along a powerline right-of-way in subalpine coniferous forest. *The Southwestern Naturalist,* 57(4), 385-392.

Tanner, D., & Perry, J. (2007). Road effects on abundance and fitness of Galapagos lava lizards (*Microlophus albemarlensis*). *Journal of Environmental Management*, 85(2), 270-278.

Thompson, S. J., Johnson, D. H., Niemuth, N. D., & Ribic, C. A. (2015). Avoidance of unconventional oil wells and roads exacerbates habitat loss for grassland birds in the North American Great Plains. *Biological Conservation*, 192, 82-90.

Torres, A., Palacín, C., Seoane, J., & Alonso, J. C. (2011). Assessing the effects of a highway on a threatened species using Before–During–After and Before–During–After-Control–Impact designs. *Biological Conservation*, 144(9), 2223-2232.

Vargas-Salinas, F., Delgado-Ospina, I., & Lopez-Aranda, F. (2011). Amphibians and reptiles killed by motor vehicles in a Sub-Andean forest in western Colombia. *Caldasia*, 33(1), 121-138.

Vargas-Salinas, F., & Amezquita, A. (2013). Traffic noise correlates with calling time but not spatial distribution in the threatened poison frog *Andinobates bombetes*. *Behaviour*, 150(6), 569-584.

Vistnes, I., & Nellemann, C. (2001). Avoidance of cabins, roads, and power lines by reindeer during calving. *The Journal of wildlife management*, 65(4), 915-925.

Ward, R. L., Anderson, J. T., & Petty, J. T. (2008). Effects of road crossings on stream and streamside salamanders. *The Journal of Wildlife Management*, 72(3), 760-771.

Wiącek, J., Polak, M., Kucharczyk, M., & Bohatkiewicz, J. (2015). The influence of road traffic on birds during autumn period: Implications for planning and management of road network. *Landscape and Urban Planning*, 114, 76-82.

Wiącek, J., & Polak, M. (2015). Does traffic noise affect the distribution and abundance of wintering birds in a managed woodland?. *Acta Ornithologica*, 50(2), 233-245.

Xu, F., Yang, W., Xu, W., Xia, C., Liao, H., & Blank, D. (2013). The effects of the Taklimakan desert highway on endemic birds *Podoces biddulph*i. *Transportation Research Part D: Transport and Environment*, 20, 12-14.

Yahner, R. H., Bramble, W. C., & Byrnes, W. R. (2001). Effect of vegetation maintenance of an electric transmission right-of-way on reptile and amphibian populations. Journal of Arboriculture, 27(1), 24-29.

Yahner, R. H., Bramble, W. C., & Byrnes, W. R. (2001). Response of amphibian and reptile populations to vegetation maintenance of an electric transmission line right-of-way. *Journal of Arboriculture*, 27(4), 215-221.

Yost, A. C., & Wright, R. G. (2001). Moose, caribou, and grizzly bear distribution in relation to road traffic in Denali National Park, Alaska. *Arctic*, 54(1), 41-48.

Van der Zande, A. N., Ter Keurs, W. J., & Van der Weijden, W. J. (1980). The impact of roads on the densities of four bird species in an open field habitat—evidence of a long-distance effect. *Biological conservation*, 18(4), 299-321.
